# Measurement of Blood Velocity With Laser Scanning Microscopy: Modeling and Comparison of Line-Scan Image-Processing Algorithms

**DOI:** 10.3389/fphys.2022.848002

**Published:** 2022-04-07

**Authors:** Emmanuelle Chaigneau, Serge Charpak

**Affiliations:** ^1^Institut de la Vision, INSERM U968, Paris, France; ^2^Institut de la Vision, CNRS UMR 7210, Paris, France; ^3^Institut de la Vision, Sorbonne Université, Paris, France

**Keywords:** laser scanning microscopy, blood velocity, image processing, line-scan, modeling, multi-photon, blood hemodynamics, vessels

## Abstract

Laser scanning microscopy is widely used to measure blood hemodynamics with line-scans in physiological and pathological vessels. With scans of broken lines, i.e., lines made of several segments with different orientations, it also allows simultaneous monitoring of vessel diameter dynamics or the activity of specific cells. Analysis of red blood cell (RBC) velocity from line-scans requires specific image-processing algorithms, as angle measurements, Line-Scanning Particle Image Velocimetry (LSPIV) or Fourier transformation of line-scan images. The conditions under which these image-processing algorithms give accurate measurements have not been fully characterized although the accuracy of measurements vary according to specific experimental parameters: the vessel type, the RBC velocity, the scanning parameters, and the image signal to noise ratio. Here, we developed mathematical models for the three previously mentioned line-scan image-processing algorithms. Our models predict the experimental conditions in which RBC velocity measurements are accurate. We illustrate the case of different vessel types and give the parameter space available for each of them. Last, we developed a software generating artificial line-scan images and used it to validate our models.

## Introduction

Blood flow dynamics is a major functional reporter of the pathophysiology of most organs. In the brain, functional hyperemia, a local increase of blood flow triggered by neuronal activation, is used as a proxy to map brain activation (Iadecola, 2017). At the cellular resolution, blood velocity measurements are commonly performed with laser scanning microscopy, either with one-photon excitation and confocal detection for superficial vessels (Dirnagl et al., 1992; Villringer et al., 1994; Zhong et al., 2008), or with multi-photon excitation for deeper vessels in tissue (Kleinfeld et al., 1998; Chaigneau et al., 2003; Shih et al., 2012; Kong et al., 2016; Kisler et al., 2018). Accurate measurement of RBC velocity with laser scanning microscopy is therefore essential for quantitative comparison of data acquired in different animals, different experimental conditions or using other techniques. RBC velocity is usually assessed with line-scans, i.e., repetitive scanning along a longitudinal portion of the central axis of a vessel. As blood velocity is mostly laminar (Rovainen et al., 1993; Logean and Riva, 2003; Zhong et al., 2011; Kornfield and Newman, 2015), RBCs usually come one after the other on the vessel axis. Consequently, line-scans produce space-time (XT) images in which each RBC leaves an oblique shadow. RBC velocity is calculated from these shadows using two types of algorithms: first, image-processing algorithms, which provide an apparent velocity of RBC (V_RBC app_); second an algorithm that takes into account the scanning velocity, and its direction relative to RBC flow, to calculate the real RBC velocity from V_RBC app_ (Chaigneau et al., 2019).

Here we focus at the first analysis step based on image-processing algorithms. The appearance of line-scan images and their processing depend on the types of vessels studied and RBC velocity. Initial studies, which were performed in capillaries where RBC velocity ranges from nearly zero to about 1 or 2 mm/s, used angle measurement either after binarization (Dirnagl et al., 1992; Villringer et al., 1994; Chaigneau et al., 2003; Zhong et al., 2008) or by application of shearing deformation on line-scan images (Kleinfeld et al., 1998). RBC velocity measurements were then extended to larger vessels, i.e., arterioles and venules, where velocity is higher and its quantification becomes a technical challenge in terms of image acquisition and analysis (Hutchinson et al., 2006; Kang et al., 2006; Shih et al., 2009; Drew et al., 2010; Rungta et al., 2018). For this reason, a variety of image processing algorithms have been developed: line fitting (Zhang et al., 2005), Radon Transform (Drew et al., 2010; Chhatbar and Kara, 2013; Joseph et al., 2019), Line-scan Particle Image Velocity (LSPIV) (Kim et al., 2012), and Fourier Transform (Autio et al., 2011; Rungta et al., 2018). All these image-processing algorithms have been successfully used, but in specific experimental conditions. Nevertheless, as vessels are tortuous and with various lengths in a focal plane, the experimental conditions rarely match the scanning conditions for which each individual image-analysis algorithm was developed. Consequently, experimenters cannot easily choose the best algorithm and the right acquisition parameters to get accurate blood velocity measurements.

Here, we developed mathematical models of the “Angle” (Dirnagl et al., 1992; Kleinfeld et al., 1998; Drew et al., 2010; Chhatbar and Kara, 2013), the LSPIV (Kim et al., 2012) and the “Fourier transform” (Autio et al., 2011; Rungta et al., 2018) algorithms to determine the conditions under which blood velocity can be measured with a chosen level of error, while simultaneously monitoring calcium signals in the neuropil. We also provide a software generating artificial line-scan images that can be used to test and validate the models for any experimental condition.

## Materials and Methods

### Definition of Variables

**FIGURE 1:**
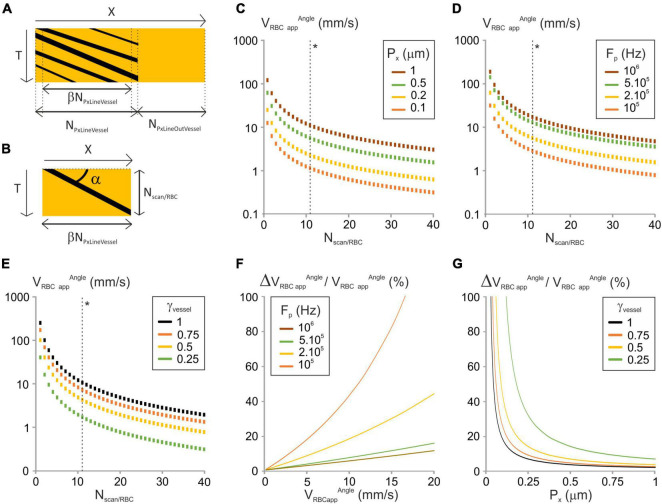
Analysis of the “Angle” image-processing algorithm. **(A)** Scheme of a typical line-scan (XT) image with its parameters. X is the distance along the scanned segment; N_PxLineVessel_ and N_PxLineOutVessel_ are the respective number of pixels of the sections within and out of the vessel, and β is the fraction of N_LineVessel_ where the scanning velocity is constant. T is the axis of time repetitions. **(B)** Principle of the “Angle” line-scan image-processing algorithm. The angle of the stripe left by the RBC (α) with the horizontal axis is used to calculate V_RBCapp_^Angle^. N_scan/RBC_ is the number of lines in which a single RBC leaves a dark shadow. **(C)** V_RBCapp_^Angle^ vs. N_scan/RBC_ was calculated from Equation (4) for a range of P_x_, with γ_Vessel_ = 1, F_p_ = 200 kHz, N_PxLineVessel_ = 50, J/(fτ) = 2 10^–8^ s^2^/m and γ_Flyback_ = 0.5. (*) The dashed line gives V_RBCapp_^Angle^ corresponding to ΔV_RBCapp/_V_RBCapp_ = 10%. **(D)** V_RBCapp_^Angle^ vs. N_scan/RBC_ was calculated from Equation (4) for a range of F_p_, with γ_Vessel_ = 1, P_x_ = 0.5 μm, N_PxLineVessel_ = 50, J/(fτ) = 2 10^–8^ s^2^/m and γ_Flyback_ = 0.5. (*) The dashed line gives V_RBCapp_^Angle^ corresponding to ΔV_RBCapp_^Angle^_/_V_RBCapp_^Angle^ = 10%. **(E)** V_RBCapp_^Angle^ vs. N_scan/RBC_ was calculated from Equation (4) for a range of γ_Vessel_, with F_p_ = 200 kHz, P_x_ = 0.5 μm, N_PxLineVessel_ = 50, J/(fτ) = 2 10^–8^ s^2^m^–1^ and γ_Flyback_ = 0.5. (*) The dashed line gives V_RBCapp_^Angle^ corresponding to ΔV_RBCapp_^Angle^_/_V_RBCapp_^Angle^ = 10%. **(F)** ΔV_RBCapp_^Angle^_/_V_RBCapp_^Angle^ vs. V_RBCapp_^Angle^ was calculated from Equation (6) for a range of F_p_ with P_x_ = 0.5 μm, N_PxLineVessel_ = 50, J/(fτ) = 2 10^–8^ s^2^/m, γ_Flyback_ = 0.5 and γ_Vessel_ = 1. **(G)** ΔV_RBCapp_^Angle^_/_V_RBCapp_^Angle^ vs. P_x_ was calculated from Equation (6) for a range of γ_Vessel_ with N_PxLineVessel_ = 50, J/(fτ) = 2 10^–8^ s^2^/m, γ_*Flyback*_ = 0.5, F_p_ = 200 kHz and V_RBCapp_ = 2 mm/s.

We considered a line-scan image where the scanned segment was made of a segment within a vessel of N_PxLineVessel_ pixels and a segment out of the vessel of N_PxLineOutVessel_ pixels (Figure 1A). This last segment can be used to collect calcium signals from the vessel wall (endothelial cells, mural cells, astrocytes) from nearby neurons, or to simultaneously measure the dynamics of the vessel diameter.

We defined the following parameters to perform our analysis: γ_Flyback_ is the fraction of time for the scanners to come back from the end of the line to the beginning of the next one over the time for the scanners to scan the broken line γ_Vessel_ is the fraction of line within the vessel:

$$\gamma_{\text{Vessel}}=\frac{N_{P\,x\,L\,i\,n\,e\,V\,e\,s\,s\,e\,l}}{N_{P\,x\,L\,i\,n\,e\,V\,e\,s\,s\,e\,l}+N_{P\,x\,L\,i\,n\,e\,O\,u\,t\,V\,e\,s\,s\,e\,l}}$$

During acquisition, experimenters also control the pixel size (P_x_) and the pixel clock (F_p_) which is the acquisition frequency of pixels. Their product gives the scanning velocity (V_scan_). Using these parameters, the time from the beginning of one line to the beginning of the next one (T_Line_) can be calculated:

$$T_{L\,i\,n\,e}=\left(1+\gamma_{F\,l\,y\,b\,a\,c\,k}\right)\,\left(N_{P\,x\,L\,i\,n\,e\,V\,e\,s\,s\,e\,l}+N_{P\,x\,L\,i\,n\,e\,O\,u\,t\,V\,e\,s\,s\,e\,l}\right)/F_{p}$$

This can be reformulated:


(1) $$T_{L\,i\,n\,e}=\left(1+\gamma_{F\,l\,y\,b\,a\,c\,k}\right)\,N_{P\,x\,L\,i\,n\,e\,V\,e\,s\,s\,e\,l}/\left(F_{p}\,\gamma_{\text{Vessel}}\right)$$

### Pre-processing of Line-Scan Images

Before analysis of RBCs velocity with the considered algorithms, line-scan images undergo two steps of preprocessing. The first one is a cropping step. Indeed, because of inertia of the scanning system, the instantaneous scanning velocity (V_scan_^inst^) does not always match the target scanning velocity, i.e., the product of the pixel size by the pixel clock. The scanning system stops at the beginning of each segment of the broken line, and then accelerates until it reaches its target velocity, and then decreases close to the end of the segment until it stops at the end. To prevent scanning velocity changes from biasing RBCs velocity measurements, images are cropped to remove the regions where the scanning velocity is not the target one (i.e., not constant). The extent of this regions can be estimated from the rotor inertia and Torque: for small angles, V_scan_ can be estimated by the product of the focal length of the objective (f) by the angular velocity of the scanning system (ω):

$$V_{s\,c\,a\,n}^{i\,n\,s\,t}\approx f\,\omega$$

The angular velocity can be calculated from the fundamental principle of dynamics, knowing the scanners inertia (J) and the rotor Torque (τ):

$$\tau =J\,\frac{d\,\omega }{d\,t}$$

Therefore, acceleration of the scanners can be estimated by:

$$\frac{d\,V_{s\,c\,a\,n}^{i\,n\,s\,t}}{d\,t}\approx \frac{f\,\tau }{J}$$

Therefore, the number of pixels that needs to be cropped at each edge of each section (N_crop_) is the ratio of the target velocity by the acceleration of the scanners normalized by the pixel clock:


(2) $$N_{c\,r\,o\,p}=\frac{P_{x}\,F_{p}^{2}\,J}{f\,\tau }$$

N_crop_ is a linear function of J/τ, which ranges from 10^–8^ s^2^ to 10^–6^ s^2^ for standard systems. Therefore, the lower the ratio J/τ is, the lower is the number of pixels that need to be cropped. N_crop_ increases with the pixel size and the pixel clock. N_crop_ is inversely proportional to the focal length of the objective which ranges from 2 to 15 mm for high NA water immersion objectives used in multiphoton microscopy. As the higher the magnification is, the lower the focal length of the objective, the higher N_crop_ is. Experimental pixel sizes range from 0.1 to 1 μm. and acquisition frequencies range from 50 kHz to 1 MHz. In many conditions, N_crop_ equals zero (Supplementary Figures 1A,B). This is the case if J/τ ≤ 8 10^–8^ s^2^ and F_p_ ≤ 200 kHz. Therefore, in these conditions, the scanners velocity is always the target one except at the points at the end of the segment. For higher pixel clocks, N_crop_ can reach tens of pixels and the variations in scanning velocity need to be considered.

The fraction of N_LineVessel_ where the scanning velocity is constant is thus:


(3) $$\beta =1-\frac{2\,N_{c\,r\,o\,p}}{N_{P\,x\,L\,i\,n\,e\,V\,e\,s\,s\,e\,l}}=1-\frac{2\,P_{x}\,F_{p}^{2}\,J}{N_{P\,x\,L\,i\,n\,e\,V\,e\,s\,s\,e\,l}\,f\,\tau }$$

The second pre-processing step is a filtering step that consists in application of filters (often median filters, that does not alter shapes) to reduce noise.

### Mathematical Modeling

Numerical simulations were performed with Microsoft Excel. γ_Flyback_ was set to 0.5 and J/τ = 3.7 10^–8^ s^2^ (galvanometer system Cambridge Technology 8315). The diameter of RBCs (D_RBC_), which varies depending on RBC orientation and vessel (Chaigneau et al., 2003), was set to 6 μm.

### Artificial Line-Scan Images

We developed a software with NI LabVIEW 2020 to generate artificial line-scan images as described in Supplementary Figures 2A,B and Supplementary Information. This software can be downloaded from GitHub: https://github.com/EmmanuelleChaigneau/Create_simulated_RBC_xtfast_Image. Using it, we generated 1.2 s long artificial line-scans of constant velocity (V_RBCapp_^image^) as in Supplementary Figure 2C. The distribution RBCs was set to be randomly varying using a flat distribution. The tube Hematocrit (H_*t*_) was set to 35% which is the average of published values: for the cortex 22, 47, and 37% (Santisakultarm et al., 2012; Lyons et al., 2016; Li et al., 2019), and 35% for the olfactory bulb (Lyons et al., 2016). White noise was added to all images so that the amplitude of noise was 1.5 times larger than the difference of amplitude between “black” pixels of RBCs and “white” pixels of plasma.

To test for our models when the RBCs flow is laminar, the number of pixels on the distance axis was set to 50 or 100, F_p_ was set to 200 kHz or 1 MHz, γ_Flyback_ was set to 0.5, V_RBCapp_^image^ ranged from 0 to 20 mm/s and V_scan_ ranged from 0 to 200 mm/s.

In arteries and venules which are large enough so that RBCs could change their radial position, the flow of RBCs is not always laminar and the trajectory of some RBCs move in or out of the imaged line. This effect can be visualized when the scanned line is very long, as in Figures 1, 2 from Drew et al. (2010). To address these conditions, we created artificial line-scans in which a fraction of RBCs get “defocused,” i.e., move in or out of the scanned line. We set V_RBCapp_^image^ to a realistic value for a small artery or venule: 5.33 mm/s. We set the scanning parameters so that RBCs velocity would be calculated with an error below 10% with each of the three considered algorithms based on our models: N_PxLineVessel_ = 120, P_x_ = 0.8 μm and *F*_p_ = 400 kHz. We applied increasing levels of RBCs defocusing for 0–100% RBCs.

### Analysis of Line-Scan Images

Each image was first pre-processed using median filters to reduce noise without altering the shape of RBCs shadows. Then pre-processed images were split into 0.1 s long sections and each section was processed by each image-analysis algorithm giving V_RBCapp_^Angle^, V_RBCapp_^LSPIV^, and V_RBCapp_*^TF^*. For the angle algorithm we used the method from Kleinfeld et al. (1998), for the LSPIV algorithm the method from Kim et al. (2012) and for the “Fourier transform” the method from Rungta et al. (2018). The first and the last sections were discarded to avoid edge effects. The relative error was calculated as (V_RBCapp_^Algorithm^–V_RBCapp_^Image^)/V_RBCapp_^Image^ for each 0.1 s long section. Then the maximum relative error over the ten computed points that was defined as: $\max_{1\leq i\leq 10}\left(V_{\text{RBCapp}}^{\mathrm{Computed}\,i}-V_{\text{RBCapp}}^{\text{Image}}\right)/V_{\text{RBCapp}}^{\text{Image}}$.

## Results

### Mathematical Models for Image Analysis Algorithms

#### Strategy Followed

For each considered image-processing algorithms, we analyzed the conditions to get the relative error on the apparent velocity of RBC below an error level (P%), and the range of apparent velocities of RBC that can be reached in these conditions. We considered RBC speeds from 0.1 mm/s which can be measured in capillaries to 20 mm/s which is reached in larger arteries. We assumed that line-scan images had been pre-processed (see Methods section “Pre-Processing of Line-Scan Images”). We investigated the impact of every parameter that can be experimentally controlled: the number of pixels within the vessel (N_PxLineVessel_), the fraction of pixels within the vessel (γ_Vessel_), the fraction of time for the scanners to come back from the end of the line to the beginning of the next one over the time for the scanners to scan the broken line (γ_Flyback_), the pixel size (P_x_) and the pixel clock (F_p_). The pixel size and the pixel clock are often merged into a single parameter: V_scan_ which is their product. Scanning velocities usually range between 20 and 100 mm/s even if they can go down to 5 mm/s and up to 200 mm/s (Kleinfeld et al., 1998; Chaigneau et al., 2003; Schaffer et al., 2006; Autio et al., 2011; Kim et al., 2012; Chhatbar and Kara, 2013; Kornfield and Newman, 2015; Dunn et al., 2018; Kisler et al., 2018).

In this manuscript we used only positive values for all velocities. When the relative directions of scanning and RBC flow needed to be considered, an extra variable was used for the sign.

#### 1st Algorithm: “Angle”

We first investigated the image-processing algorithms where the apparent velocity is calculated from the angle (α) between the shadows left by RBCs and the distance axis (Figure 1B) (Dirnagl et al., 1992; Kleinfeld et al., 1998; Drew et al., 2010; Chhatbar and Kara, 2013). This angle is measured either directly (Dirnagl et al., 1992) or by application of shear stress to the line-scan image until the shadows left by RBCs appear to be horizontal (Kleinfeld et al., 1998). The apparent velocity is the inverse of tangent of α scaled by the units on the horizontal and the vertical axes of the line-scan image, respectively, P_x_ and T_Line_:

$$V_{R\,B\,C\,a\,p\,p}^{A\,n\,g\,l\,e}=\frac{P_{x}}{T_{L\,i\,n\,e}\,\tan\alpha }$$

Tan α can be calculated from the number of pixels on the adjacent and opposite sides of the α angle:

$$\tan\alpha =\frac{N_{S\,c\,a\,n/R\,B\,C}}{\beta \,N_{P\,x\,L\,i\,n\,e\,V\,e\,s\,s\,e\,l}}$$

where N_Scan/RBC_ + 1 is the number of scanned lines on which the RBC appears on the cropped line-scan image (Figure 1B). Using equations (1) and (3):


(4) $$V_{R\,B\,C\,a\,p\,p}^{A\,n\,g\,l\,e}=\frac{\left(1-\frac{2\,P_{x}\,F_{p}^{2}\,J}{N_{P\,x\,L\,i\,n\,e\,V\,e\,s\,s\,e\,l}\,f\,\tau }\right)\,\gamma_{\text{Vessel}}\,P_{x}\,F_{p}}{\left(1+\gamma_{F\,l\,y\,b\,a\,c\,k}\right)\,N_{S\,c\,a\,n/R\,B\,C}}$$

N_Scan/RBC_ takes integer values due to line-scan image pixelation, so V_RBCapp_^Angle^ can only take discrete values (Figures 1C–E). V_RBCapp_^Angle^ increases with P_x_, F_p_ and γ_Vessel_ (Figures 1C–E). Therefore, these parameters may need to be increased to reach the largest apparent velocities of RBCs.

The error on V_RBCapp_^Angle^ (ΔV_RBCapp_^Angle^) is set by the acquired line-scan image pixelation and corresponds to an error of one line in estimating N_Scan/RBC_:

$$△\,V_{R\,B\,C\,a\,p\,p}^{A\,n\,g\,l\,e}=\frac{\left(1-\frac{2\,P_{x}\,F_{p}^{2}\,J}{N_{P\,x\,L\,i\,n\,e\,V\,e\,s\,s\,e\,l}\,f\,\tau }\right)\,\gamma_{\text{Vessel}}\,P_{x}\,F_{p}}{\left(1+\gamma_{F\,l\,y\,b\,a\,c\,k}\right)\,\left(N_{S\,c\,a\,n/R\,B\,C}-1\right)}-\frac{\left(1-\frac{2\,P_{x}\,F_{p}^{2}\,J}{N_{P\,x\,L\,i\,n\,e\,V\,e\,s\,s\,e\,l}\,f\,\tau }\right)\,\gamma_{\text{Vessel}}\,P_{x}\,F_{p}}{\left(1+\gamma_{F\,l\,y\,b\,a\,c\,k}\right)\,N_{\frac{S\,c\,a\,n}{R\,B\,C}}}=\frac{V_{R\,B\,C\,a\,p\,p}^{A\,n\,g\,l\,e}}{\left(N_{S\,c\,a\,n/R\,B\,C}-1\right)}$$

The relative error on the apparent RBC velocity is thus:


(5) $$\frac{\Delta \,V_{R\,B\,C\,a\,p\,p}^{A\,n\,g\,l\,e}}{V_{R\,B\,C\,a\,p\,p}^{A\,n\,g\,l\,e}}=\frac{1}{\left(N_{S\,c\,a\,n/R\,B\,C}-1\right)}$$

This can be reformulated:


(6) $$\frac{△\,V_{R\,B\,C\,a\,p\,p}^{A\,n\,g\,l\,e}}{V_{R\,B\,C\,a\,p\,p}^{A\,n\,g\,l\,e}}=\frac{\left(1+\gamma_{F\,l\,y\,b\,a\,c\,k}\right)\,V_{R\,B\,C\,a\,p\,p}^{A\,n\,g\,l\,e}}{\left(1-\frac{2\,P_{x}\,F_{p}^{2}\,J}{N_{P\,x\,L\,i\,n\,e\,V\,e\,s\,s\,e\,l}\,f\,\tau }\right)\,\gamma_{\text{Vessel}}\,P_{x}\,F_{p}-\left(1+\gamma_{F\,l\,y\,b\,a\,c\,k}\right)\,V_{R\,B\,C\,a\,p\,p}^{A\,n\,g\,l\,e}}$$

With the “angle” algorithm, the relative error on RBC apparent velocity increases with V_RBC app_^Angle^ (Figure 1F). Thus, setting a maximum error level on the relative error on RBC apparent velocity will set an upper limit on the apparent velocity of RBCs. In addition, the relative error on the apparent velocity of RBCs decreases when P_x_, F_p_ or γ_Vessel_ increase (Figures 1F,G). Consequently, the larger P_x_, F_p_ or γ_Vessel_ are, the lower the error is. So having part of the broken line out of the vessel will reduce the accuracy of measurements.

From Equation (5), to get the relative error on RBC apparent velocity below P%, we need:

N_*Scan*RBC_ ≥ 1 + 100/P.

Therefore, the maximal measurable apparent velocity allowing the relative error on RBC apparent velocity below P% is:


(7) $$V_{R\,B\,C\,a\,p\,p\,\max P\%}^{A\,n\,g\,l\,e}=\frac{\left(1-\frac{2\,P_{x}\,F_{p}^{2}\,J}{N_{P\,x\,L\,i\,n\,e\,V\,e\,s\,s\,e\,l}\,f\,\tau }\right)\,\gamma_{\text{Vessel}}\,P_{x}\,F_{p}}{\left(1+\frac{100}{P}\right)\,\left(1+\gamma_{F\,l\,y\,b\,a\,c\,k}\right)}$$

V_RBCapp_^Angle^_*maxP*%_ has thus the same dependence to scanning parameters as V_RBCapp_^Angle^ (Figures 1C–E dashed lines next to (*)).

#### 2nd Algorithm: LSPIV

**FIGURE 2:**
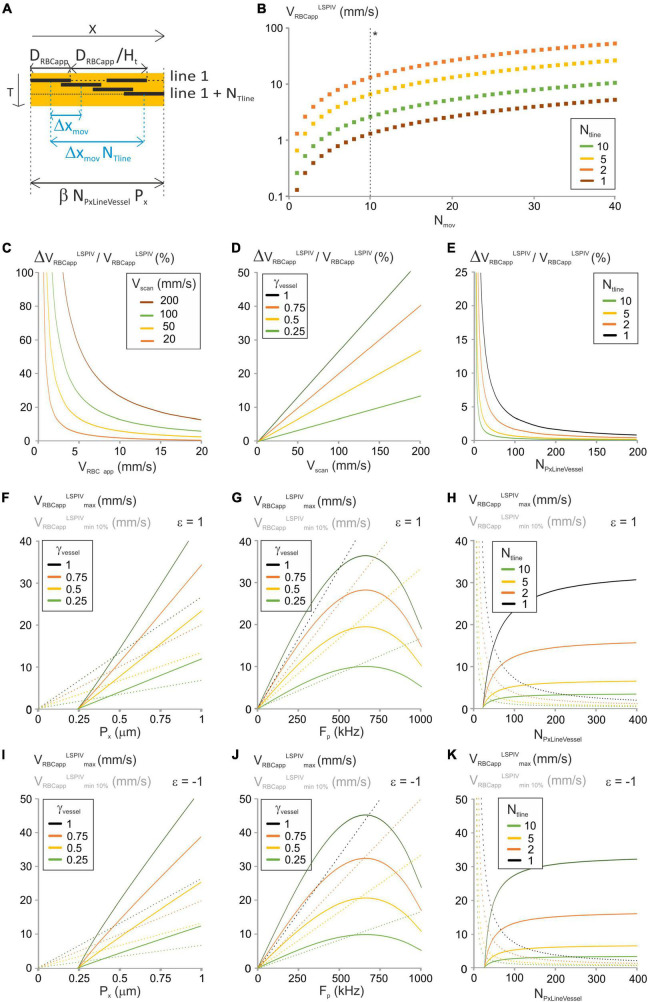
Analysis of the “LSPIV” image-analysis method. **(A)** Principle of the “LSPIV” image processing algorithm and specific parameters considered in the modeling. D_RBC*app*_ is the size of the shadow left by an RBC, and H_*t*_ is hematocrit. ΔX_mov_ is the displacement of a RBC from one line to the successive one. Two lines from the spatiotemporal image separated by N_Tline_ lines are selected. Their intensities are then cross-correlated and the maximum of the cross-correlogram is found, giving N_Tline_ ΔX_mov_. V_RBC app_ is the ratio of ΔX_mov_ by T_Line_. **(B)** V_RBCapp_^LSPIV^ vs. N_mov_ was calculated from Equation (8) for a range of N_Tline_ with γ_Vessel_ = 1, N_PxLineVessel_ = 50, γ_Flyback_ = 0.5 and V_scan_ = 100 mm/s. (*) The dashed line gives V_RBCapp_^LSPIV^. **(C)** ΔV_RBCapp_^LSPIV^/V_RBCapp_^LSPIV^ vs. V_RBCapp_^LSPIV^ was calculated from Equation (10) for a range of V_scan_ with γ_Vessel_ = 1, N_*PxLine*Vessel_ = 50, γ_Flyback_ = 0.5 and N_Tline_ = 1. **(D)** ΔV_RBCapp_^LSPIV^/V_RBCapp_^LSPIV^ vs. V_scan_ was calculated from Equation (10) for a range of γ_Vessel_ with V_RBCapp_ = 5 mm/s, N_*PxLine*Vessel_ = 50, γ_Flyback_ = 0.5 and N_Tline_ = 1. **(E)** ΔV_RBCapp_^LSPIV^/V_RBCapp_^LSPIV^ vs. N_PxLineVessel_ was calculated from Equation (10) for a range of N_Tline_ with V_RBCapp_ = 5 mm/s, V_scan_ = 100 mm/s, γ_Flyback_ = 0.5 and γ_Vessel_ = 1. **(F)** V_RBCapp_^LSPIV^_*minP*%_ and V_RBCapp_^LSPIV^_*max*_ were calculated vs. P_x_ from Equation (11) and Equation (13), respectively, for a range of γ_Vessel_ with forward scanning, P = 10%, N_PxLineVessel_ = 50, Fp = 200 kHz, J/(fτ) = 2 10^–8^ s^2^/m, γ_Flyback_ = 0.5 and N_Tline_ = 1. **(G)** V_RBCapp_^LSPIV^_*minP*%_ and V_RBCapp_^LSPIV^_*max*_ were calculated vs. F_p_ from Equation (11) and Equation (13), respectively, for a range of γ_Vessel_ with forward scanning, P = 10%, N_PxLineVessel_ = 50, P_x_ = 0.5 μm, J/(fτ) = 2 10^–8^ s^2^/m, γ_*Flyback*_ = 0.5 and N_Tline_ = 1. **(H)** V_RBCapp_^LSPIV^_*minP*%_ and V_RBCapp_^LSPIV^_*max*_ were calculated vs. N_PxLineVessel_ from Equation (11) and Equation (13), respectively, for a range of N_Tline_ with forward scanning, P = 10%, F_p_ = 200 kHz, P_x_ = 0.5 μm, J/(fτ) = 2 10^–8^ s^2^/m, γ_Flyback_ = 0.5 and γ_Vessel_ = 1. **(I–K)** Respectively as **(F–H)** for reverse scanning.

We then investigated the LSPIV image analysis method from Kim et al. (2012). With LSPIV, two rows from the spatio-temporal image, which correspond to two recordings of fluorescence over the broken line starting at two time-points, are selected and cross-correlated to determine the displacement of RBCs from one recording to the other (Figure 2A). The apparent velocity of RBCs is then calculated as the ratio of this displacement by the time separating the two rows. The two rows can be successive or more distant, hence a new parameter N_Tline_, which correspond to the number of lines separating them. N_Tline_ = 1 corresponds to successive lines. The cross-correlation is performed in the Fourier space.

Defining Δx_mov_ as the displacement of RBCs from one line to the successive one. The values that Δx_mov_ can take depend on the choice of N_Tline._ Indeed, the cross-correlation step gives the product N_Tline._ Δx_mov_ which needs to be a multiple of the pixel size. Calling N_mov_ this number of pixels:

$$N_{T\,l\,i\,n\,e}\,\Delta \,X_{m\,o\,v}=N_{m\,o\,v}\,P_{x}$$

Therefore:

$$\Delta \,X_{m\,o\,v}=\frac{N_{m\,o\,v}\,P_{x}}{N_{T\,l\,i\,n\,e}}$$

If N_Tline_ = 1, Δx_mov_ needs to be a multiple of the pixel size, but if N_Tline_ > 1, Δx_mov_ takes values which are fractions of the pixel size. The apparent velocity is calculated as:

$$V_{R\,B\,C\,a\,p\,p}^{L\,S\,P\,I\,V}=\frac{N_{T\,l\,i\,n\,e}\,\Delta \,X_{m\,o\,v}}{N_{T\,l\,i\,n\,e}\,T_{l\,i\,n\,e}}=\frac{\Delta \,X_{m\,o\,v}}{T_{l\,i\,n\,e}}$$

Therefore:


(8) $$V_{R\,B\,C\,a\,p\,p}^{L\,S\,P\,I\,V}=\frac{\gamma_{\text{Vessel}}\,N_{m\,o\,v}\,V_{s\,c\,a\,n}}{\left(1+\gamma_{F\,l\,y\,b\,a\,c\,k}\right)\,N_{T\,l\,i\,n\,e}\,N_{P\,x\,L\,i\,n\,e\,V\,e\,s\,s\,e\,l}}$$

Here, the pixel size and the pixel clock can be merged in a single parameter: the scanning velocity. Therefore, they have the same influence on RBCs velocity measurements. N_mov_ and N_Tline_ take only integer values due to line-scan image pixelation, so V_RBC app_^LSPIV^ can only take discrete values (Figure 2B). Increasing N_Tline_ allow reaching lower values of the apparent velocity. The error on V_RBCapp_^LSPIV^ is set by image pixelization within a row and can therefore be calculated by replacing N_mov_ by 1 in equation (8):


(9) $$△\,V_{R\,B\,C\,a\,p\,p}^{L\,S\,P\,I\,V}=\frac{\gamma_{\text{Vessel}}\,V_{s\,c\,a\,n}}{\left(1+\gamma_{F\,l\,y\,b\,a\,c\,k}\right)\,N_{T\,L\,i\,n\,e}\,N_{P\,x\,L\,i\,n\,e\,V\,e\,s\,s\,e\,l}}$$

Hence:


(10) $$\frac{△\,V_{R\,B\,C\,a\,p\,p}^{L\,S\,P\,I\,V}}{V_{R\,B\,C\,a\,p\,p}^{L\,S\,P\,I\,V}}=\frac{\gamma_{\text{Vessel}}\,V_{s\,c\,a\,n}}{\left(1+\gamma_{F\,l\,y\,b\,a\,c\,k}\right)\,N_{T\,L\,i\,n\,e}\,N_{P\,x\,L\,i\,n\,e\,V\,e\,s\,s\,e\,l}\,V_{R\,B\,C\,a\,p\,p}^{L\,S\,P\,I\,V}}$$

With LSPIV, the relative error on the apparent velocity of RBCs decreases when the apparent velocity of RBCs increases (Figure 2C). Thus, setting a maximum error level will set a lower limit on V_RBCapp_^LSPIV^. Furthermore ΔV_RBCapp_^LSPIV^/V_RBCapp_^LSPIV^ increases linearly with V_scan_ (Figures 2C,D). Consequently, V_scan_ needs to be reduced to measure accurately the lower values of V_RBCapp_^LSPIV^. The relative error on the apparent velocity of RBCs increases with γ_Vessel_ too (Figure 2D). Therefore, using a line comprising a segment out of the vessel increases the accuracy of LSPIV measurements. In addition, the relative error on the apparent velocity of RBCs is inversely proportional to N_Tline_ and N_PxLineVessel_ (Figure 2E). Therefore, choosing lines composed of many pixels and increasing N_Tline_ during analysis increases the possibility to measure accurately lower values of V_RBCapp_^LSPIV^.

To get ΔV_RBCapp_^LSPIV^/V_RBCapp_^LSPIV^ ≤ *p*%, we need:

$$\frac{100\,\gamma_{\text{Vessel}}\,V_{s\,c\,a\,n}}{P\,\left(1+\gamma_{F\,l\,y\,b\,a\,c\,k}\right)\,N_{T\,L\,i\,n\,e}\,N_{P\,x\,L\,i\,n\,e\,V\,e\,s\,s\,e\,l}}\leq V_{R\,B\,C\,a\,p\,p}^{L\,S\,P\,I\,V}$$

Thus, the minimum apparent RBC velocity that can be measured is:


(11) $$V_{R\,B\,C\,a\,p\,p\,\min P\%}^{L\,S\,P\,I\,V}=\frac{100\,\gamma_{V\,e\,s\,s\,e\,l}\,V_{s\,c\,a\,n}}{P\,\left(1+\gamma_{F\,l\,y\,b\,a\,c\,k}\right)\,N_{T\,L\,i\,n\,e}\,N_{P\,x\,L\,i\,n\,e\,V\,e\,s\,s\,e\,l}}$$

It corresponds to N_mov_ = 100/P in equation (8). It varies with V_scan_, i.e., to the pixel size and acquisition frequency, with γ_Vessel_, with N_Tline_ and N_PxLineVessel_ as the relative error does (Figures 2F–H). Consequently, the lower the velocities of interest, the smaller pixel size, acquisition frequency and γ_Vessel_ are needed, and choosing lines composed of many pixels and increasing N_Tline_ during analysis is necessary.

Velocity measurements can be accurate only if the cross-correlation is accurate. We estimated that a minimum of 2 shadows is needed for a successful cross-correlation. Accounting for the plasma around them at the chosen hematocrit (H_*t*_) level (Figure 2A), the length of broken line that can be used for cross correlation needs to be larger than2 D_RBCapp_/H_*t*_, where D_RBCapp_ is the size of the shadow left by a RBC and is given by Chaigneau et al. (2019):

$$D_{R\,B\,C\,a\,p\,p}=D_{R\,B\,C}\,\left(1+\varepsilon \,\frac{V_{R\,B\,C\,a\,p\,p}}{V_{s\,c\,a\,n}}\right)$$

D_RBC_ is the real size of a RBC, with unsigned values for V_scan_ and V_RBCapp_, and ε = 1 if scanning and RBC flow are oriented in the same direction or ε = −1 if scanning and RBC flow are opposite.

Therefore:

$$\beta \,N_{P\,x\,L\,i\,n\,e\,V\,e\,s\,s\,e\,l}\,P_{x}\geq \frac{2\,D_{R\,B\,C}\,\left(1+\varepsilon \,\frac{V_{R\,B\,C\,a\,p\,p}^{L\,S\,P\,I\,V}}{V_{s\,c\,a\,n}}\right)}{H_{t}}$$

Using equation (3):

$$\left(1-\frac{2\,P_{x}\,F_{p}^{2}\,J}{N_{P\,x\,L\,i\,n\,e\,V\,e\,s\,s\,e\,l}\,f\,\tau }\right)\,N_{P\,x\,L\,i\,n\,e\,V\,e\,s\,s\,e\,l}\,P_{x}\geq \frac{2\,D_{R\,B\,C}\,\left(1+\varepsilon \,\frac{V_{R\,B\,C\,a\,p\,p}^{L\,S\,P\,I\,V}}{P_{x}\,F_{p}}\right)}{H_{t}}$$

Distributing and rearranging, this gives a condition on the number of pixels within the vessel:


(12) $$N_{P\,x\,L\,i\,n\,e\,V\,e\,s\,s\,e\,l}\geq \frac{2\,D_{R\,B\,C}}{H_{t}\,P_{x}}\,\left(1+\varepsilon \,\frac{V_{R\,B\,C\,a\,p\,p}^{L\,S\,P\,I\,V}}{P_{x}\,F_{p}}\right)+\frac{2\,P_{x}\,F_{p}^{2}\,J}{f\,\tau }$$

The largest apparent RBC velocity that can be measured with the LSPIV algorithm ($V_{\mathrm{RBCapp}\,\max}^{\text{LSPIV}}$) is set by possibility to follow RBC shadows during cross-correlation. It is the ratio of the maximum distance 2 RBCs can move within a line, by the time between the cross-correlated lines:

$$V_{R\,B\,C\,a\,p\,p\,m\,a\,x}^{L\,S\,P\,I\,V}=\frac{\frac{\beta \,N_{P\,x\,L\,i\,n\,e\,V\,e\,s\,s\,e\,l}\,P_{x}-2\,D_{R\,B\,C\,a\,p\,p}}{2}}{N_{T\,L\,i\,n\,e}\,T_{L\,i\,n\,e}}$$

This can be reformulated (see Supplementary Information 1.1):


(13) $$V_{R\,B\,C\,a\,p\,p\,m\,a\,x}^{L\,S\,P\,I\,V}=\frac{\gamma_{\text{Vessel}}\,P_{x}\,\left(N_{P\,x\,L\,i\,n\,e\,V\,e\,s\,s\,e\,l}\,P_{x}\,F_{p}-\frac{2\,P_{x}^{2}\,F_{p}^{3}\,J}{f\,\tau }-2\,D_{R\,B\,C}\,F_{p}\right)}{2\,\left(1+\gamma_{F\,l\,y\,b\,a\,c\,k}\right)\,P_{x}\,N_{T\,L\,i\,n\,e}\,N_{P\,x\,L\,i\,n\,e\,V\,e\,s\,s\,e\,l}+2\,\varepsilon \,\gamma_{\text{Vessel}}\,D_{R\,B\,C}}$$

The largest apparent RBC velocity that can be measured with the LSPIV algorithm increases with the pixel size (Figures 2F,I), but it has a bell-shaped dependency to the acquisition frequency, first increasing, then reaching a maximum before decreasing (Figures 2G,J). Therefore, in the range of experimental values for the pixel size and the acquisition frequency, the inertia term has only an impact when varying the acquisition frequency. Reducing γ_Vessel_ reduces $V_{\mathrm{RBCapp}\,\max}^{\text{LSPIV}}$ (Figures 2F,G) and thus could impact the possibility to measure the largest velocities. $V_{\mathrm{RBCapp}\,\max}^{\text{LSPIV}}$ is also larger when scanning is opposite to RBC flow (Figures 2I–K), increasing the range of parameters giving accurate measurements. Therefore, it is beneficial to scan opposite to the RBC flow. The maximum apparent RBC velocity that can be measured increases with N_PxLineVessel_ (Figures 2H,K) but decreases when N_Tline_ increases. Therefore, choosing lines composed of many pixels and decreasing N_Tline_ during analysis is necessary to reach the highest values of $V_{\text{RBCapp}}^{\text{LSPIV}}$.

The same logic gives another constrain on the minimum N_*Px*LineVessel_ to reach V_RBCapp_^LSPIV^. Indeed, the length of the line within the vessel that can be used to compute velocities needs to be large enough to fit the shadow of two RBCs and their movement over N_Tline_ lines at V_RBCapp_^LSPIV^ (Figure 2A):

$$\beta \,N_{P\,x\,L\,i\,n\,e\,V\,e\,s\,s\,e\,l}\,{\text{P}}_{x}\geq 2\,\left({\text{N}}_{T\,l\,i\,n\,e}\,{\text{T}}_{l\,i\,n\,e}\,{\text{V}}_{R\,B\,C\,a\,p\,p}^{L\,S\,P\,I\,V}+{\text{D}}_{R\,B\,C\,a\,p\,p}\right)$$

This can be reformulated (see details in Supplementary Information 1.2):


(14) $$N_{P\,x\,L\,i\,n\,e\,V\,e\,s\,s\,e\,l}\geq \frac{2\,\gamma_{\text{Vessel}}\,D_{R\,B\,C}\,\left(P_{x}\,F_{p}+\varepsilon \,V_{R\,B\,C\,a\,p\,p}^{L\,S\,P\,I\,V}\right)+\frac{2\,\gamma_{\text{Vessel}}\,P_{x}^{3}\,F_{p}^{3}\,J}{f\,\tau }}{\gamma_{\text{Vessel}}\,P_{x}^{2}\,F_{p}-2\,\left(1+\gamma_{F\,l\,y\,b\,a\,c\,k}\right)\,N_{T\,L\,i\,n\,e}\,P_{x}\,V_{R\,B\,C\,a\,p\,p}^{L\,S\,P\,I\,V}}$$

And $N_{T\,L\,i\,n\,e}\leq \frac{\gamma_{\text{Vessel}}\,P_{x}\,F_{p}}{2\,\left(1+\gamma_{F\,l\,y\,b\,a\,c\,k}\right)\,V_{R\,B\,C\,a\,p\,p}^{L\,S\,P\,I\,V}}$

#### 3rd Algorithm: Fourier Transform of Image

**FIGURE 3:**
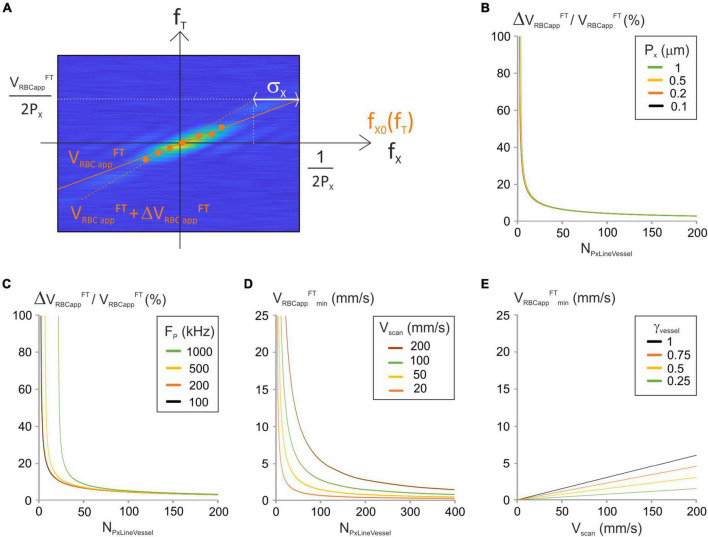
Analysis of the “Fourier-transform” image-analysis method. **(A)** Principle of the “Fourier Transform” image-processing algorithm: modulus of a Fourier-transformed spatiotemporal image (magnified for the purpose of clarity) with time frequencies (f_*T*_), spatial frequencies (f_x_), maximal spatial frequency F_*x max*_ = 1/(2P_x_), expectancy of the spatial frequencies f_x0_(f_Ti_) for some temporal frequencies (f_Ti_) (orange dots) and standard deviation of the spatial frequencies (σ_x_). The relation between f_x0_(f_Ti_) and f_Ti_ is fit by a first order polynomial whose first order coefficient is V_RBCapp_*^FT^*(orange full line). Its upper point was shifted by σ_x_ to get V_RBC app_^FT^ + ΔV_RBC app_^FT^ (orange dashed line). **(B)** ΔV_RBC app_^FT^_/_V_RBC app_^FT^ was calculated vs. N_PxLineVessel_ from Equation (18) for a range of P_x_ with F_p_ = 200 kHz and J/(fτ) = 2 10^–8^ s^2^/m. **(C)** ΔV_RBC app_^FT^_/_V_RBC app_^FT^ was calculated vs. N_PxLineVessel_ from Equation (18) for a range of F_p_ with P_x_ = 0.5 μm and J/(fτ) = 2 10^–8^ s^2^/m. **(D)** V_RBC app_^FT^_min_ vs. N_PxLineVessel_ was calculated from Equation (20) for a range of V_scan_ with γ_Vessel_ = 1 and γ_Flyback_ = 0.5. **(E)** V_RBC app_^FT^_min_ vs. V_scan_ was calculated from Equation (20) for a range of γ_Vessel_ with N_PxLineVessel_ = 50 and γ_Flyback_ = 0.5.

We last modeled the conditions to use the Fourier Transform image analysis method (Autio et al., 2011; Rungta et al., 2018). This method uses the 2D Fourier Transform of the line-scan image to calculate the apparent velocity of RBCs. The 2D Fourier Transform of the line-scan image can be described by a series of distributions. At each time frequency (f_Ti_) can be associated a distribution of spatial frequencies f_x_(f_Ti_). The 2D Fourier Transform of the line-scan image is the sum of these distributions: $\sum_{i}f_{\text{x}}\left(f_{Ti}\right)$ (Figure 3A). The apparent velocity is calculated in two steps: First for each time frequency f_Ti_, the expected value f_x0_(f_Ti_) of the spatial frequencies f_x_(f_*Ti*_) is calculated. Then the relation between f_x0_(f_Ti_) and f_Ti_ is fit by a first order polynomial whose first order coefficient is V_RBCapp_^FT^.

Accurate calculation of spatial frequencies requires that the length of broken line used to calculate the spatial frequencies is large enough to fit two RBC shadows and the plasma in between them at the chosen hematocrit level (Figure 2A). This expression was already calculated (Equation (12)) for the LSPIV algorithm:


(15) $$N_{P\,x\,L\,i\,n\,e\,V\,e\,s\,s\,e\,l}\geq \frac{2\,D_{R\,B\,C}}{H_{t}\,P_{x}}\,\left(1+\varepsilon \,\frac{V_{R\,B\,C\,a\,p\,p}^{L\,S\,P\,I\,V}}{P_{x}\,F_{p}}\right)+\frac{2\,P_{x}\,F_{p}^{2}\,J}{f\,\tau }$$

The major source of error on V_RBCapp_^FT^ is due to estimation of f_x0_(f_Ti_). ΔV_RBCapp_^FT^ can thus be estimated by a displacement by σ_x_, the standard deviation of f_x0_(f_Ti_), at the limiting spatial frequency which is Nyquist frequency 1/(2P_x_) (Figure 3A). The corresponding temporal frequency is: $\frac{{\text{V}}_{\mathrm{RBCapp}}^{\text{FT}}}{2\,{\text{P}}_{x}}=\left({\text{V}}_{\mathrm{RBCapp}}^{\text{FT}}+\Delta \,{\text{V}}_{\mathrm{RBCapp}}^{\text{FT}}\right)\,\left(\frac{1}{2\,{\text{P}}_{x}}-\sigma_{x}\right)$

Therefore:

$$\Delta \,{\text{V}}_{\mathrm{RBCapp}}^{F\,T}=\frac{{\text{V}}_{\mathrm{RBCapp}}^{F\,T}}{\left(\frac{1}{2\,\sigma_{x}\,{\text{P}}_{x}}-1\right)}$$

σ_x_ can be calculated from the distribution of f_x_(f_Ti_) whose probability density function is (Rungta et al., 2018):

$$\beta \,N\,\text{PxLineVessel}\,P_{x}\,s\,i\,n\,c^{2}\,\left(\pi \,\beta \,N\,\text{PxLineVessel}\,P_{x}\,\left(f_{x}\,\left({\text{f}}_{T\,i}\right)-\text{f}\,\mathrm{x0}\,\left(f\,\text{Ti}\right)\right)\right)$$

Where sinc stands for the sinus cardinal function, which is the ratio of the sinus function by the identity function.

This gives (details in Supplementary Information 1.3):


(16) $$\sigma_{x}\approx \frac{1}{2\,P_{x}\,\pi \,{\left(\frac{N_{P\,x\,L\,i\,n\,e\,V\,e\,s\,s\,e\,l}}{2}-\frac{P_{x}\,F_{p}^{2}\,J}{f\,\tau }\right)}^{1/2}}$$

The error on the apparent velocity ΔV_RBCapp_^FT^ can now be expressed as a function of V_RBCapp_^FT^:


(17) $$\Delta \,{\text{V}}_{\mathrm{RBCapp}}^{F\,T}\approx \frac{{\text{V}}_{\mathrm{RBCapp}}^{F\,T}}{\pi \,{\left(\frac{N_{P\,x\,L\,i\,n\,e\,V\,e\,s\,s\,e\,l}}{2}-\frac{P_{x}\,F_{p}^{2}\,J}{f\,\tau }\right)}^{1/2}-1}$$

And


(18) $$\frac{\Delta \,{\text{V}}_{\mathrm{RBCapp}}^{F\,T}}{{\text{V}}_{\mathrm{RBCapp}}^{F\,T}}\approx \frac{1}{\pi \,{\left(\frac{N_{P\,x\,L\,i\,n\,e\,V\,e\,s\,s\,e\,l}}{2}-\frac{P_{x}\,F_{p}^{2}\,J}{f\,\tau }\right)}^{1/2}-1}$$

From this expression, the relative apparent velocity depends on N_PxLineVesse*l*_, P_x_, F_p_ and the ratio of the scanner inertia over the rotor Torque. The relative apparent velocity decreases when N_*PxLine*VesseI_ increases (Figures 3B,C). Therefore, the higher N_PxLineVessel_ is the more accurate V_RBCapp_^FT^ is. The impact of the pixel size on the relative apparent velocity is limited (Figure 3B) but reducing the acquisition frequency allows to use lower values of N_PxLineVesseI_ (Figure 3C).

To get the relative error on the apparent RBC velocity below P%, we need:


(19) $${\text{N}}_{P\,x\,L\,i\,n\,e\,V\,e\,s\,s\,e\,l}\geq 2\,\left({\left(\frac{100+P}{\pi \,P}\right)}^{2}+\frac{P_{x}\,F_{p}^{2}\,J}{f\,\tau }\right)$$

The minimum velocity is set by pixelation of the modulus of the Fourier Transformed spatiotemporal image (Figure 3A). It is the ratio of the pixel size of the temporal frequency axis by the maximal spatial frequency:

$${\text{V}}_{\mathrm{RBC}\,\mathrm{app}\,\min}^{F\,T}=\frac{\frac{1}{T_{l\,i\,n\,e}}}{\frac{1}{2\,P_{x}}}$$

This can be reformulated:


(20) $${\text{V}}_{\mathrm{RBCappmin}}^{F\,T}=\frac{2\,\gamma_{\text{Vessel}}\,{\text{V}}_{\text{scan}}}{\left(1+\gamma_{\text{Flyback}}\right)\,{\text{N}}_{P\,x\,L\,i\,n\,e\,V\,e\,s\,s\,e\,l}}$$

Again F_p_ and P_x_ can be merged into a single parameter: the scanning velocity. The minimum velocity is proportional to V_scan_ and γ_Vessel_ and inversely proportional to N_PxLineVesseI_. Therefore, measurement of low RBC velocities requires using either low scanning velocities, or many pixels per line, or a line comprising a portion out of the vessel (Figures 3D,E).

### Parameter Space Ensuring Accurately Velocity Measurement Over a Given Range

Using these models, it is now possible to determine the image-analysis method and the experimental parameters which ensure that the error on apparent velocities measured is within the range of accepted values.

#### 1st Algorithm: Angle

For the “angle” method, conditions on N_PxLineVessel_, P_x_ and F_p_, are induced by the maximal measurable apparent velocity allowing an apparent speed error below P% (Equation (7)). To measure V_RBCapp_ ≤ V_RBCapp max P%_, we need (details in Supplementary Information 1.4):


(21) $$N_{P\,x\,L\,i\,n\,e\,V\,e\,s\,s\,e\,l}\geq \frac{2\,\gamma_{\text{Vessel}}\,P_{x}^{2}\,F_{p}^{3}\,J}{f\,\tau \,\left(\gamma_{\text{Vessel}}\,P_{x}\,F_{p}-\left(1+\frac{100}{P}\right)\,\left(1+\gamma_{F\,l\,y\,b\,a\,c\,k}\right)\,V_{R\,B\,C\,a\,p\,p}^{A\,n\,g\,l\,e}\right)}$$

And


(22) $$P_{x}\geq \frac{\left(1+\frac{100}{P}\right)\,\left(1+\gamma_{\text{Flyback}}\right)\,V_{R\,B\,C\,a\,p\,p}}{\gamma_{\text{Vessel}}\,F_{p}}$$

Together, Equations (21) and (22) allow to calculate the parameter space for the “angle” algorithm.

#### 2nd Algorithm: LSPIV

For the LSPIV method, the parameter space is given by several equations: the minimum number of pixels necessary to use the LSPIV method is set by Equations (12) and (14) calculated before. Other constraints on N_PxLineVessel_ come from Equation (11), which gives the minimal measurable apparent velocity allowing an error below P%. To measure V_RBCapp_^LSPIV^ ≥ V_RBCapp_^LSPIV^_min P%_, we need:


(23) $${\text{N}}_{P\,x\,L\,i\,n\,e\,V\,e\,s\,s\,e\,l}\geq \frac{100\,\gamma_{\text{Vessel}}\,{\text{V}}_{\text{scan}}}{\left(1+\gamma_{\text{Flyback}}\right)\,P\,N_{T\,L\,i\,n\,e}\,{\text{V}}_{\mathrm{RBCapp}}^{L\,S\,P\,I\,V}}$$

Together, Equations (12), (14), and (23) allow to calculate the parameter space for the LSPIV algorithm.

#### 3rd Algorithm: Fourier Transform of Image

For the Fourier transform method, the parameter space is also given by several equations. Accurate calculation of spatial frequencies requires that the length of broken line used to calculate the spatial frequencies is large enough to fit two RBC shadows and the plasma surrounding them at the chosen hematocrit level which gives Equation (15). Another condition comes from the necessity to keep the relative error on the apparent RBC velocity below P%, which is given by equation (19). In addition to that, a condition on N_*PxLine*Vessel_ comes from the minimum velocity from Equation (20). To ensure that V_RBCapp_^FT^ ≥ V_RBCapp_^FT^_min,_ it is necessary that:


(24) $${\text{N}}_{P\,x\,L\,i\,n\,e\,V\,e\,s\,s\,e\,l}\geq \frac{2\,\gamma_{\text{Vessel}}\,{\text{V}}_{\text{scan}}}{\left(1+\gamma_{\text{Flyback}}\right)\,{\text{V}}_{\mathrm{RBCapp}}^{F\,T}}$$

Together, Equations (15), (19), and (24) allow to calculate the parameter space for the Fourier Transform algorithm.

#### Parameter Space of Typical Vessels

**FIGURE 4:**
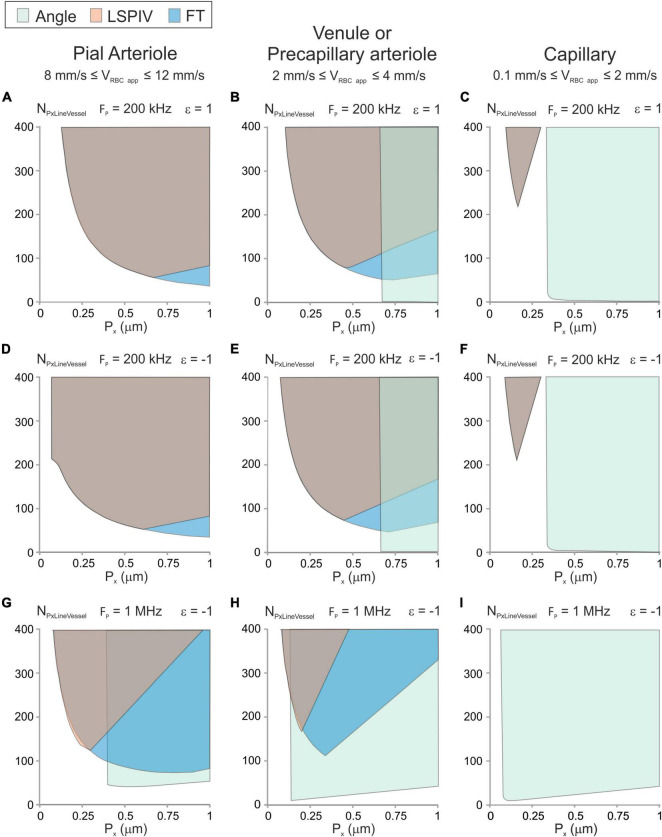
Parameter space and image-analysis algorithms that can be used with typical vessel types. Graphic showing the possible experimental conditions (P_x_, N_PxLineVessel_) and image-analysis algorithms for typical vessels calculated from Equations (12), (14), (15), (21)–(24) for an error level of 10% with D_RBC_ = 6 μm, H_*t*_ = 0.35 and γ_Vessel_ = 0.5. **(A–C)** Anterograde scanning and F_p_ = 200 kHz. **(D–F)** Retrograde scanning and F_p_ = 200 kHz. **(G–I)** Retrograde scanning and F_p_ = 1 MHz. The parameter space satisfying the conditions to get accurate velocity measurement is colored in green for the “Angle” algorithm, in orange for the LSPIV algorithm and in blue for the Fourier Transform algorithm. For some combinations of scanning parameters, colors are overlaid. **(A,D,G)** Example of a parenchymal arteriole with 8 mm/s ≤ V_RBC app_ ≤ 12 mm/s. **(B,E,H)** Example of a venule or a precapillary arteriole with 2 mm/s ≤ V_RBC app_ ≤ 4 mm/s. **(C,F,I)** Example of a capillary with 0.1 mm/s ≤ V_RBC app_ ≤ 1 mm/s.

The scanning parameter space can be drawn using Equations (12), (14), (15), (21), (22), (23) and (24) (Methods section “Mathematical Modeling” and Figure 4). We considered 3 types of vessels: A pial arteriole with a large velocity (8 mm/s ≤ V_RBC app_ ≤ 12 mm/s), a venule or a precapillary arteriole with 2 mm/s ≤ V_RBC app_ ≤ 4 mm/s and a capillary with small velocity (0.1 mm/s ≤ V_RBC app_ ≤ 2 mm/s). The parameter space was investigated for anterograde and retrograde scanning, for various pixel clocks. For every type of vessel, using retrograde scanning was beneficial as it enlarged the parameter space for both the LSPIV and the FT algorithms (Figures 4A,B vs. Figures 4D,E).

For the pial arteriole (Figures 4A,D,G), the “Angle” algorithm can only be used with the largest pixel clock and large pixel sizes, which correspond to large scanning velocities. The LSPIV algorithm and the Fourier Transform algorithm can both be used over broad parameter spaces which overlap nearly completely for the lowest pixel clocks.

The parameter space of the venule or precapillary arteriole (Figures 4B,E,H) shows that the three image-processing algorithms can be used but over different regions of the parameter space. The “Angle” algorithm can be used for the large scanning velocities. The LSPIV algorithm can be used over a broad parameter space and the Fourier Transform algorithm can be used over a parameter space even larger, which includes the parameter space of the LSPIV algorithm.

The parameter space of the capillary (Figures 4C,F,I) shows that the “Angle” algorithm can be used over a large region of the parameter space for every pixel clock. It is the most adapted image-processing algorithm for capillaries. The LSPIV algorithm and the Fourier Transform algorithms can be used too for the lowest pixel clock but only over the same small region, with small pixel sizes, which correspond to small and very large number of pixels within the line.

For each type of vessel, there is a region of the parameter space where none of the studied image-processing algorithms can be used.

### Tests of the Three Image-Processing Algorithms With Artificial Line-Scans

We last controlled the validity of our models using artificial line-scans of known constant velocity. We processed images using the three studied algorithms and compared computed velocities to the image velocity. We had to use artificial line-scans as the velocity of real line-scan images would be unknown, making comparison with computed velocities impossible. For this purpose, we developed a software generating artificial line-scan images (described in Supplementary Information) and used it to get images of constant velocity ($V_{\mathrm{RBC}\,\mathrm{app}}^{\text{Image}})$ in various scanning conditions as described in section “Materials and Methods.” Artificial line-scans were analyzed with each image-processing algorithm, as described in “Materials and Methods,” giving the relative error of each algorithm for each artificial line-scan.

**FIGURE 5:**
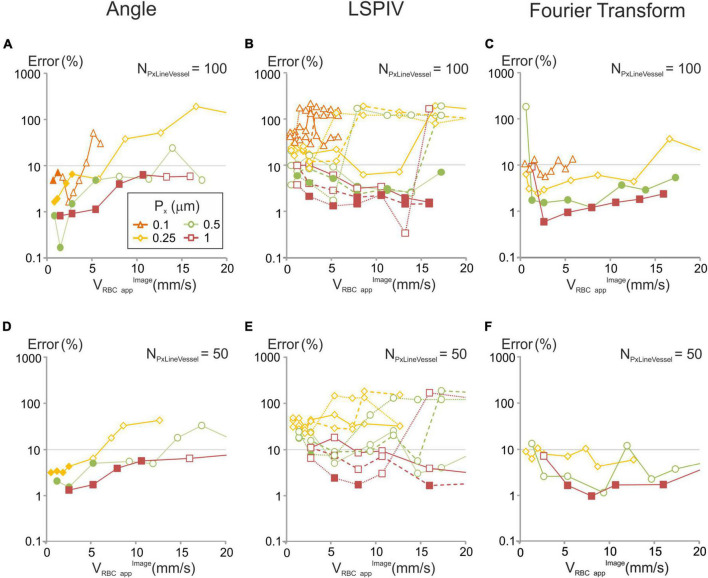
Errors in velocities computed with each of the image-analysis algorithms for various of acquisition parameters and comparison with our models for a 10% error level. Artificial line-scans of constant velocity (V_RBCapp_^image^) were processed with each algorithm: **(A,D)** angle; **(B,E)** LSPIV, full line (N_Tline_ = 1), dashed lines (N_Tline_ = 2) and dotted lines (N_Tline_ = 5); **(C,F)** Fourier Transform, and the relative error was calculated (see methods) in two scanning conditions: **(A–C)** N_PxLineVessel_ = 100, **(D–F)** N_PxLineVessel_ = 50, with F_p_ = 200 kHz. When the scanning conditions met the criteria in which our model predicts a signal over noise be-low 10%, a full symbol was used. In other conditions an empty symbol is used.

The relative error was compared to the predictions of our models for a 10% error level. For each algorithm, for every combination of scanning parameters satisfying the conditions defined by our models, the relative error of the corresponding algorithm was within the chosen level of accuracy (Figure 5).

Finally, we examined the accuracy of RBCs velocity measurements in situations where the flow of RBCs is not perfectly laminar, and the trajectory of some RBCs get “defocused”: i.e., move in or out of the imaged line (see the example in Figures 1, 2). We created artificial line-scans with increasing levels of RBCs defocusing (see Methods section “Artificial Line-Scan Images”) and set the scanning parameters so that, in the in absence of RBCs defocusing, V_RBCapp_ would be calculated with an error below 10% with each of the three considered algorithms based on our models. We applied the three studied algorithms on these images and compared computed velocities to the image velocity (Supplementary Figures 3B–D). RBCs defocusing did not impact a lot the error on RBC velocity measurements. With the “angle” algorithm, it increased only when all RBCs defocused. With the LSPIV algorithm and the Fourier transform algorithms, it increased slightly. In every case, the error remained below 10%. Therefore, RBCs defocusing has little impact on our model predictions to get accurate velocity measurements.

## Discussion

### Importance of Accurate Velocity Measurements

Laser scanning imaging is a technique of choice to investigate blood flow dynamics in wild type animals (Kleinfeld et al., 1998; Chaigneau et al., 2003; Shih et al., 2012; Schmid et al., 2019) and in several models of disease, as diverse as Alzheimer’s disease (Kisler et al., 2017), HIV (Silva et al., 2014), cancer (Brown et al., 2001; Kamoun et al., 2010; Santisakultarm et al., 2014), traumatic brain injury (Bragin et al., 2017), ischemia (Shih et al., 2009; Ishikawa et al., 2016; McConnell et al., 2016; Liu et al., 2018), or inflammation (Honkura et al., 2018). RBC velocity measurements are not limited to the brain (Santisakultarm et al., 2014; Silva et al., 2014; Ishikawa et al., 2016; McConnell et al., 2016; Bragin et al., 2017; Liu et al., 2018) becoming common for the kidney (Kang et al., 2006; Dunn et al., 2018), the retina (Kornfield and Newman, 2015), the skin (Brown et al., 2001), the hindlimb (Lasch et al., 2018), the mammary fat pad (Kamoun et al., 2010), or the ear (Honkura et al., 2018) in various conditions. This stresses that, to compare velocity data acquired in various experimental conditions, within and in between laboratories, both at resting state and in response to any physiological stimulus, accurate measurements are paramount, particularly if velocity differences amount to as little as a few percent.

### Our Models

Our models are nearly exclusively based on experimental parameters. For the LSPIV model, we imposed that two RBCs are scanned per line to get a successful cross-correlation, mostly because when a single RBC shadow appears on one line and two shadows appear on the next line, the cross-correlation has significant chances to be incorrect. This assumption proved true when running the LSPIV algorithm with artificial line-scans. On a wider scale, analysis of artificial line-scans has shown that in the scanning conditions defined by each of our models, the accuracy of the computed velocities always matched the defined criteria, showing that our models are sufficient to get accurate measurements.

### Parameter Space and Image-Analysis Algorithms

Our models show that each image-processing algorithm has a specific dependence on each scanning parameter and this specificity needs to be taken into account to perform accurate measurements. RBC velocity is a determining criterion. Among the algorithms we modeled, the “Angle” algorithm (Dirnagl et al., 1992; Kleinfeld et al., 1998) is the most suitable to measure velocity in capillaries whereas the LSPIV (Kim et al., 2012) and Fourier Transform (Rungta et al., 2018) algorithms are the most suitable for large artery measurements. In intermediate range of velocities, each algorithm is suitable over a specific parameter space that needs to be calculated. The parameter space often comprises a region where no algorithm is suitable. Note that retrograde scanning is always advisable as it enlarges the parameter space, particularly in the large range of RBC velocities.

## Conclusion

Overall, the combination of our new software providing artificial line-scan images and the mathematical models for the “Angle” algorithm, the LSPIV and the Fourier Transform image-processing algorithms allow vascular biologists to choose the right algorithm and the experimental parameters that will give RBC velocity values with a chosen level of accuracy. This will enable unbiased and accurate comparisons of blood hemodynamic parameters in control and pathological animal models.

## Data Availability Statement

The datasets presented in this study can be found in an online repository: https://github.com/EmmanuelleChaigneau/Create_simulated_RBC_xtfast_Image.

## Author Contributions

EC designed the project, developed the mathematical models, developed the software, analyzed the data, and wrote the manuscript. SC designed the project, wrote the manuscript, and supervised the project. Both authors contributed to the article and approved the submitted version.

## Conflict of Interest

The authors declare that the research was conducted in the absence of any commercial or financial relationships that could be construed as a potential conflict of interest.

## Publisher’s Note

All claims expressed in this article are solely those of the authors and do not necessarily represent those of their affiliated organizations, or those of the publisher, the editors and the reviewers. Any product that may be evaluated in this article, or claim that may be made by its manufacturer, is not guaranteed or endorsed by the publisher.

## Abbreviations

β: Fraction of N_LineVessel_ where the scanning velocity is constant
D_RBC_: size of an RBC
D_RBCapp_: size of the shadow left by an RBC over the line-scan image along the distance axis
Δ x_mov_: displacement of a RBC from one line to the successive one
ε: variable used to characterize the relative orientation of RBC flow and scanning. ε equals 1 if scanning and RBC flow are oriented in the same direction and ε equals − 1 if scanning and RBC flow are opposite
f: focal length of the objective
F_p_: acquisition frequency of pixels
H_t_: Hematocrit
γ_Flyback_: fraction of forward scanning time needed for the scanner to scan back go back from the end of the line to the beginning of the next one
γ_Vessel_: fraction of line within the vessel
J: scanner inertia
N_mov_: number of pixels by which RBCs move from one row of the line-scan image to the next one
N_PxLineVessel_: number of pixels per line within the vessel
N_Scan/RBC_: number of scanned lines on which the RBC appears minus one
N_Tline_: number of lines separating the two lines cross-correlated with the LSPIV algorithm
P_x_: pixel size
RBC: Red Blood Cell
Tau: rotor Torque
T_Line_: scanning time from the beginning of one line to the beginning of the next one
V_scan_: scanning velocity.
