# Supplementary material for: Measurement of Blood Velocity With Laser Scanning Microscopy: Modeling and Comparison of Line-Scan Image-Processing Algorithms
Source: Front Physiol. 2022 Apr 7;13:848002. doi: 10.3389/fphys.2022.848002 (PMC9022085; doi:10.3389/fphys.2022.848002)
Supplement: Supplementary file 1 [file Data_Sheet_1.pdf]

Measurement of blood velocity with laser scanning microscopy: Modelling and comparison of line-scan image-processing algorithms.

Emmanuelle CHAIGNEAU and Serge CHARPAK

Supplementary Material

1 Supplementary equations

We provide here detailed development of some equations

1.1 Equation (13):

$$V_{RBCapp\,max}^{LSPIV} = \frac{\frac{\beta\, N_{PxLineVessel}\, P_x - 2\, D_{RBCapp}}{2}}{N_{TLine}\, T_{Line}}$$

Using equations (1) and (3):

$$V_{RBCapp\,max}^{LSPIV} = \frac{\left(\frac{\left(1 - \frac{2\, P_x\, F_p^2\, J}{N_{PxLineVessel}\, f\, \tau}\right) N_{PxLineVessel}\, P_x}{2} - D_{RBC}\left(1 + \varepsilon \frac{V_{RBCapp\,max}^{LSPIV}}{V_{scan}}\right)\right) F_p\, \gamma_{\text{Vessel}}}{(1+\gamma_{Flyback})\, N_{TLine}\, N_{PxLineVessel}}$$

Developing:

$$V_{RBCapp\,max}^{LSPIV} = \gamma_{\text{Vessel}} \frac{N_{PxLineVessel}\, P_x\, F_p - \frac{2\, P_x^2\, F_p^3\, J}{f\, \tau} - 2\, D_{RBC}\, F_p}{2\,(1+\gamma_{Flyback})\, N_{TLine}\, N_{PxLineVessel}} - \frac{\varepsilon\, \gamma_{\text{Vessel}}\, D_{RBC}\, V_{RBCapp\,max}^{LSPIV}}{(1+\gamma_{Flyback})\, P_x\, N_{TLine}\, N_{PxLineVessel}}$$

Grouping terms depending on $V_{RBCapp\,max}^{LSPIV}$:

$$V_{RBCapp\,max}^{LSPIV}\left(1 + \frac{\varepsilon\, \gamma_{\text{Vessel}}\, D_{RBC}}{(1+\gamma_{Flyback})\, P_x\, N_{TLine}\, N_{PxLineVessel}}\right) = \gamma_{\text{Vessel}} \frac{N_{PxLineVessel}\, P_x\, F_p - \frac{2\, P_x^2\, F_p^3\, J}{f\, \tau} - 2\, D_{RBC}\, F_p}{2\,(1+\gamma_{Flyback})\, N_{TLine}\, N_{PxLineVessel}}$$

Developing and simplifying:

$$V_{RBCapp\,max}^{LSPIV}\left(\left(1 + \gamma_{Flyback}\right) P_x\, N_{TLine}\, N_{PxLineVessel} + \varepsilon\, \gamma_{\text{Vessel}} D_{RBC}\right) = \frac{\gamma_{\text{Vessel}}\, P_x\left(N_{PxLineVessel}\, P_x\, F_p - \frac{2\, P_x^2\, F_p^3\, J}{f\, \tau} - 2\, D_{RBC}\, F_p\right)}{2}$$

Therefore:

$$V_{RBCapp\,max}^{LSPIV} = \frac{\gamma_{\text{Vessel}}\, P_x\left(N_{PxLineVessel}\, P_x\, F_p - \frac{2\, P_x^2\, F_p^3\, J}{f\, \tau} - 2\, D_{RBC}\, F_p\right)}{2(1+\gamma_{Flyback})\, P_x\, N_{TLine}\, N_{PxLineVessel} + 2\, \varepsilon\, \gamma_{\text{Vessel}} D_{RBC}} \qquad (13)$$

1.2 Equation (14):

$$\beta N_{PxLineVessel}\, P_x \geq 2\,(N_{Tline}\, T_{line} V_{RBCapp}^{LSPIV} + D_{RBCapp})$$

Developing:

$$\left(1 - \frac{2\, P_x\, F_p^2\, J}{N_{PxLineVessel}\, f\, \tau}\right) N_{PxLineVessel}\, P_x \geq \frac{2\,(1+\gamma_{Flyback})\, N_{TLine}\, N_{PxLineVessel}\, P_x\, V_{RBCapp}^{LSPIV}}{\gamma_{\text{Vessel}}\, V_{scan}} + 2\, D_{RBC}\left(1 + \varepsilon \frac{V_{RBCapp}^{LSPIV}}{V_{scan}}\right)$$

Re-arranging:

$$N_{PxLineVessel}\, P_x - \frac{2\, P_x^2\, F_p^2\, J}{f\, \tau} \geq \frac{2\,(1+\gamma_{Flyback})\, N_{TLine}\, N_{PxLineVessel}\, V_{RBCapp}^{LSPIV}}{\gamma_{\text{Vessel}}\, F_p} + 2\, D_{RBC}\left(1 + \varepsilon \frac{V_{RBCapp}^{LSPIV}}{P_x\, F_p}\right)$$

Grouping terms depending on $N_{PxLineVessel}$:

$$N_{PxLineVessel}\left(P_x - \frac{2\,(1+\gamma_{Flyback})\, N_{TLine}\, V_{RBCapp}^{LSPIV}}{\gamma_{\text{Vessel}}\, F_p}\right) \geq 2\, D_{RBC}\left(1 + \varepsilon \frac{V_{RBCapp}^{LSPIV}}{P_x\, F_p}\right) + \frac{2\, P_x^2\, F_p^2\, J}{f\, \tau}$$

Therefore:

$$N_{PxLineVessel} \geq \frac{2\, D_{RBC}\left(1 + \varepsilon \frac{V_{RBCapp}^{LSPIV}}{P_x\, F_p}\right) + \frac{2\, P_x^2\, F_p^2\, J}{f\, \tau}}{P_x - \frac{2\,(1+\gamma_{Flyback})\, N_{TLine}\, V_{RBCapp}^{LSPIV}}{\gamma_{\text{Vessel}}\, F_p}}$$

$$N_{PxLineVessel} \geq \frac{2\gamma_{\text{Vessel}}\, D_{RBC}\,(P_x\, F_p + \varepsilon\, V_{RBCapp}^{LSPIV}) + \frac{2\,\gamma_{\text{Vessel}}\, P_x^3\, F_p^3\, J}{f\, \tau}}{\gamma_{\text{Vessel}}\, P_x^2\, F_p - 2\,(1+\gamma_{Flyback})\, N_{TLine}\, P_x\, V_{RBCapp}^{LSPIV}} \tag{14}$$

And

$$\beta\, \gamma_{\text{Vessel}}\, P_x\, F_p - 2\,(1 + \gamma_{Flyback})\, N_{TLine}\, V_{RBCapp}^{LSPIV} \geq 0$$

Which can be reformulated

$$N_{TLine} \leq \frac{\beta\, \gamma_{\text{Vessel}}\, P_x\, F_p}{2\,(1 + \gamma_{Flyback})\, V_{RBCapp}^{LSPIV}}$$

1.3 Equation (16):

σ_x can be calculated from the distribution of $f_x(f_{Ti})$ whose probability density function is:

$$\beta\, N_{PxLineVessel}\, P_x sinc^2(\pi\, \beta\, N_{PxLineVessel}\, P_x\, (f_x(f_{Ti}) - f_{x0}(f_{Ti})))$$

$$\sigma_x^2 = \beta\, N_{PxLineVessel}\, P_x \int_{\frac{-1}{2P_x}}^{\frac{1}{2P_x}} (f_x - f_{x0}(f_{Ti}))^2 sinc^2(\pi\, \beta\, N_{PxLineVessel}\, P_x (f_x(f_{Ti}) - f_{x0}(f_{Ti})))\, df_x(f_{Ti})$$

Where the sinc function is defined by:

$$sinc(x) = \frac{\sin x}{x}$$

Developing the sinc function:

$$\sigma_x^2 = \frac{1}{\pi^2 \beta \text{ N}_{\text{PxLineVessel}} P_x} \int_{\frac{-1}{2P_x}}^{\frac{1}{2P_x}} sin^2(\pi \beta \text{ N}_{\text{PxLineVessel}} P_x (f_x(\text{f}_{Ti})\text{-}f_{x0}(\text{f}_{Ti})))\text{d}f_x(\text{f}_{Ti})$$

Reformulating the $\sin^2$ function:

$$\sigma_x^2 = \frac{1}{2 \pi^2 \beta \text{ N}_{\text{PxLineVessel}} P_x} \int_{\frac{-1}{2P_x}}^{\frac{1}{2P_x}} 1 - \cos(2 \pi \beta \text{ N}_{\text{PxLineVessel}} P_x (f_x(\text{f}_{Ti})\text{-}f_{x0}(\text{f}_{Ti})))\, \text{d}f_x(\text{f}_{Ti})$$

The integral can now be calculated:

$$\sigma_x^2 = \frac{1}{2 \pi^2 \beta \text{ N}_{\text{PxLineVessel}} P_x} \left[(f_x(\text{f}_{Ti}) - \frac{\sin(2 \pi \beta \text{N}_{\text{PxLineVessel}} P_x (f_x(\text{f}_{Ti})\text{-}f_{x0}(\text{f}_{Ti})))}{2\pi \beta \text{ N}_{\text{PxLineVessel}} P_x} \right]_{\frac{-1}{2P_x}}^{\frac{1}{2P_x}}$$

$$\sigma_x^2 = \frac{1}{2 \pi^2 \beta \text{ N}_{\text{PxLineVessel}} {P_x}^2} (1 - \frac{\sin(2 \pi \beta \text{ N}_{\text{PxLineVessel}}) \cos\left(2 \pi \beta \text{ N}_{\text{PxLineVessel}} P_x f_{x0}(\text{f}_{Ti})\right)}{\pi \beta \text{ N}_{\text{PxLineVessel}}})$$

$$\frac{\sin(2 \pi \beta \text{ N}_{\text{PxLineVessel}}) \cos\left(2 \pi \beta \text{ N}_{\text{PxLineVessel}} P_x f_{x0}(\text{f}_{Ti})\right)}{\pi \beta \text{ N}_{\text{PxLineVessel}}} << 1$$

Therefore

$$ {\sigma_x}^2 \approx \frac{1}{2\pi^2 \beta \text{ N}_{PxLineVessel} {P_x}^2}$$

Thus

$$\boldsymbol{\sigma_x} \approx \frac{\mathbf{1}}{\boldsymbol{\pi} \boldsymbol{P_x} (\mathbf{2} \boldsymbol{\beta} \text{ }\mathbf{N}_{\boldsymbol{PxLineVessel}})^{\mathbf{1/2}}}$$

Using equation (3):

$$\boldsymbol{\sigma_x} \approx \frac{\mathbf{1}}{\boldsymbol{\pi} \boldsymbol{P_x} (\mathbf{2} \left(\boldsymbol{N_{PxLineVessel}} - \frac{\mathbf{2} \boldsymbol{P_x} \boldsymbol{F_p^2} \boldsymbol{J}}{\boldsymbol{f} \boldsymbol{\tau}} \right))^{\mathbf{1/2}}} \qquad (16)$$

1.4 Equation (21):

$$V_{RBCapp}^{Angle} \max P\% = \frac{\left(1 - \frac{2 P_x F_p^2 J}{N_{PxLineVessel} f \tau}\right) \gamma_{\text{Vessel}} P_x F_p}{(1+\frac{100}{P}) \, (1+\gamma_{Flyback})}$$

Thus, to measure $V_{RBCapp} \leq V_{RBCapp\ max\ P\%}$, we need: $V_{RBCapp}^{Angle} \leq \frac{\left(1 - \frac{2 P_x F_p^2 J}{N_{PxLineVessel} f \tau}\right) \gamma_{Vessel} P_x F_p}{(1+\frac{100}{P})(1+\gamma_{Flyback})}$

This can be reformulated

$$\frac{(1 + \frac{100}{P})(1 + \gamma_{Flyback}) V_{RBCapp}^{Angle}}{\gamma_{Vessel} P_x F_p} \leq 1 - \frac{2 P_x F_p^2 J}{N_{PxLineVessel} f \tau}$$

This can be reformulated

$$\frac{2 P_x F_p^2 J}{N_{PxLineVessel} f \tau} \leq \frac{\gamma_{Vessel} P_x F_p - (1 + \frac{100}{P})(1 + \gamma_{Flyback}) V_{RBCapp}^{Angle}}{\gamma_{Vessel} P_x F_p}$$

Therefore

$$\frac{2 \gamma_{Vessel} P_x^2 F_p^3 J}{f \tau (\gamma_{Vessel} P_x F_p - \left(1+\frac{100}{P}\right)(1+\gamma_{Flyback}) V_{RBCapp}^{Angle})} \leq N_{PxLineVessel} \quad (21)$$

And

$$(\gamma_{Vessel} P_x F_p - \left(1 + \frac{100}{P}\right)(1 + \gamma_{Flyback}) V_{RBCapp}^{Angle} > 0$$

Which can be reformulated

$$\boldsymbol{P_x} \geq \frac{\left(1+\frac{100}{P}\right)(1+\gamma_{Flyback})\, V_{RBCapp}}{\gamma_{Vessel}\ F_p} \quad (22)$$

2 Software providing artificial line-scan images.

We have developped a software generating realistic artificial line-scan images based on user-defined parameters using NI LabVIEW 2020. This software can be downloaded from GitHub : https://github.com/EmmanuelleChaigneau/Create_simulated_RBC_xtfast_Image. Here are the parameters controled by the user (Supplementary Figure 2(A)) :

- *"Image horizontal size"* (in pixels)
- *"Image vertical size"* (in pixels)
- *"Scanning frequency"* (kHz / pixel) used to image
- *"Flyback ratio"*, fraction of forward scanning time needed for the scanner to scan back go back from the end of the line to the beginning of the next one.
- *"Pixel size"* (in µm)
- *"Target RBC velocity"* (mm/s)
- *"RBC flow direction"* which gives the direction of the RBC flow relative to the scanning direction
- *"Ht"* is the tube hematocrit (Gould and Linninger, 2015)
- *"Random / regular"*, describes the distrubution of RBCs over time. "*Regular*" means that the frequency of RBCs is constant over time, whereas "*Random*" means that their instantaneous frequency is random.
- *"Plasma level"* is the pixel value used for plasma in the absence of noise

- *“RBC level”*, pixel value used for RBCs in the absence of noise
- *“Noise”* (%), ratio of the pixel range used for noise by the difference between *“Plasma level”* and *“RBC level”*.
- “*Defocusing RBCs (%)*” is the ratio of RBCs that undergo defocusing, i.e., whose trajectory deviates from the scanned lines. Defocusing is modelled by intensity changes following a squared sinusoidal function of fixed period (the “*Defocusing period*”) and random phase. The amplitude of the defocusing is the range of pixel intensities from the RBC level to the plasma level.
- “*Defocusing period (ms)*” is the period of the squared sinusoidal function used to defocus RBCs.

The software has a state structure described on Supplementary Figure 2(B). User-defined inputs are used to calculate image parameters. Then the software tests if the image parameters are valid or not. If they are, the user can generate an image and save it. Otherwise the user can change the user inputs until they are valid.

2.1 Evaluation of image parameters:

The following image parameters are computed by the software from the user inputs:

- The “Time / line” (T_{line}) is calculated as : $T_{line} = H_{Im} (1+ \gamma_{Flyback}) / F_{scan}$, where F_{scan} is the scanning frequency, H_{Im} is the “Image horizontal size” and $\gamma_{Flyback}$ is the “Flyback ratio”.
- The “scanning velocity” (V_{scan}) is calculated as : $V_{scan} = P_x\ F_{scan}$, where P_x is the pixel size.
- *“Delta x”* (Δx_{mov}) is the displacement in pixels of a RBC from one line to the next one (Chaigneau et al., 2019): $\Delta x_{mov} = \mathrm{Round}\ [T_{line} V^T_{RBC} / (P_x (1+ \varepsilon V^T_{RBC} / V_{scan}))]$.

  Where V^T_{RBC} is the *“Target RBC velocity”* and ε is used to characterize the relative orientation of RBC flow and scanning. ε = 1 if scanning and RBC flow are oriented in the same direction and ε = -1 if scanning and RBC flow are opposite. To take into account pixelization, *“Delta x”* was rounded to the nearest integer. As a consequence, the real RBC velocity of the image does not correspond to the *“Target RBC velocity”*.
- The *“Apparent RBC velocity”* was calculated from *“Delta x”* as (Chaigneau et al., 2019): $V_{RBCapp} = \Delta X_{mov}\ P_x / T_{line}$.
- The *“Real RBC velocity”* was calculated as: $V_{RBC} = V_{RBCapp} / (1 + \varepsilon\ V_{RBCapp} / V_{scan}))$.
- “Lines / RBC” (L_{RBC}) is the number of linescans necessary so that the RBC has moved by a distance equal to its diameter. Therefore it can be calculated as the ratio of the RBC diameter (D_{RBC}) by V_{RBC} and by T_{line} : $L_{RBC} = \mathrm{Round}\ [D_{RBC} / (V_{RBC}\ T_{line})]$. To take into account pixelization, “Lines / RBC” was rounded to the nearest integer.
- “Lines / plasma” (L_{Plasma}) defines the number of plasma lines in between 2 succesive RBCs for a regular distribution of RBCs. “Lines / plasma” depends on hematocrit and “Lines / RBC”:
  $L_{Plasma} = \mathrm{Round}\ [(100 – Ht)\ L_{RBC} / Ht]$.

  To take into account pixelization, “Lines / plasma” was rounded to the nearest integer.
- “*RBC width*” is the number of pixels defining an RBC shadow on a single line. It is the product of *“Delta x”* by “*Lines / RBC*”.

2.2 Control of image parameters.

Then our software then performs a step of control of “Image parameters” which ends up with their validation or rejection.

It controls that :

- The target velocity is inferior to the scanning velocity,
- “*Delta x*” is positive and inferior to the image horizontal size,
- “*RBC width*” is inferior to the image horizontal size,
- “*Lines / plasma*” and “*Lines / RBC*” are positive,
- pixel intensity values resulting from the chosen image parameters remain within the U16 range.

2.3 Image generation.

To create noiseless images, each image pixel was given a value (F_0) correponding either to F_{Plasma}, the value of pixels in the plasma in the absence of noise, or F_{RBC}, the value of pixels in RBCs in the absence of noise. F_{Plasma} and F_{RBC} are defined by theusers.

To create a noisy image, the value of each pixel from the noiseless image was changed by adding a random number multiplied by a noise level (c), which is defined as : $F = F_0 + (\varepsilon - 0.5)\, c\, (F_{Plasma} - F_{RBC}) / 50$, where F_0 is the value of each pixel in the absence of noise, ε is a random number which has a uniform distribution between 0 and 1, c is the noise level. If c equals 100, the amplitude of the noise distribution equals $F_{Plasma} - F_{RBC}$.

To avoid any meaningless data points in images, tests are performed to ensure that F remains positive and within the data type range : $Fmax > F_0 + (\varepsilon - 0.5)\, c\, (F_{Plasma} - F_{RBC}) / 50 > 0 \;\; \forall \varepsilon$.

F reaches its maximum for ε = 1 and $F_0 = F_{Plasma}$, therefore : $Fmax > F_{Plasma} + c\, (F_{Plasma} - F_{RBC}) / 100$

F reaches its minimum for ε = 0 and $F_0 = F_{RBC}$, therefore : $F_{RBC} - c\, (F_{Plasma} - F_{RBC}) / 100 > 0$.

3 References

Chaigneau, E., Roche, M., and Charpak, S. (2019). Unbiased Analysis Method for Measurement of Red Blood Cell Size and Velocity With Laser Scanning Microscopy. *Front Neurosci* 13**,** 644.

Gould, I.G., and Linninger, A.A. (2015). Hematocrit distribution and tissue oxygenation in large microcirculatory networks. *Microcirculation* 22**,** 1-18.

4 Supplementary figures

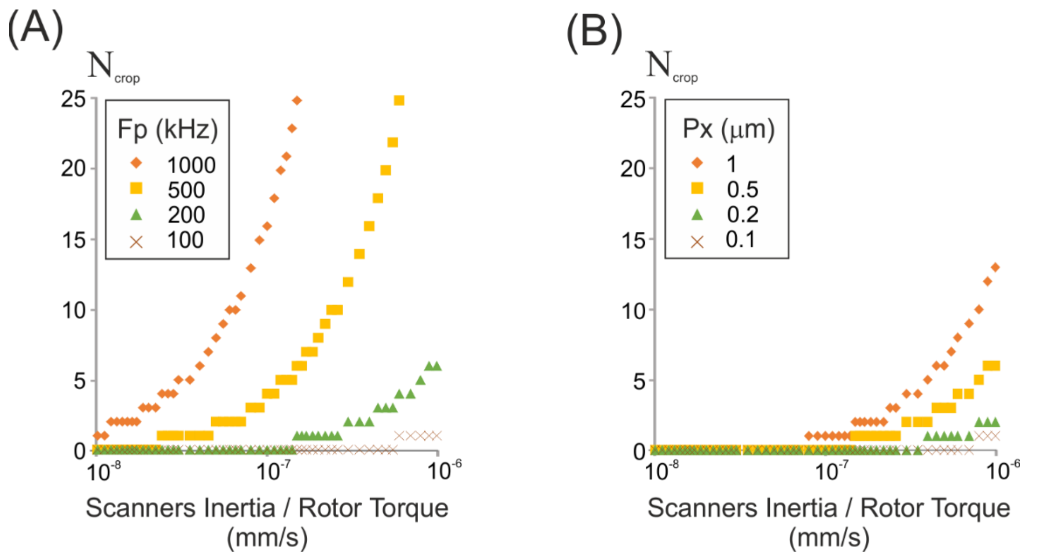

Supplementary Figure 1 : Effects Scanners inertia and rotor Torque on Line-scan images

(A) - Number of pixels that need to be croped at each edge of the section of the line-scan image within the vessel for a pixel size of 0.5 µm and for a range of pixel clocks (F_P).

(B) – Same as (A) for a pixel clock of 200 kHz and a range of pixel sizes (P_x).

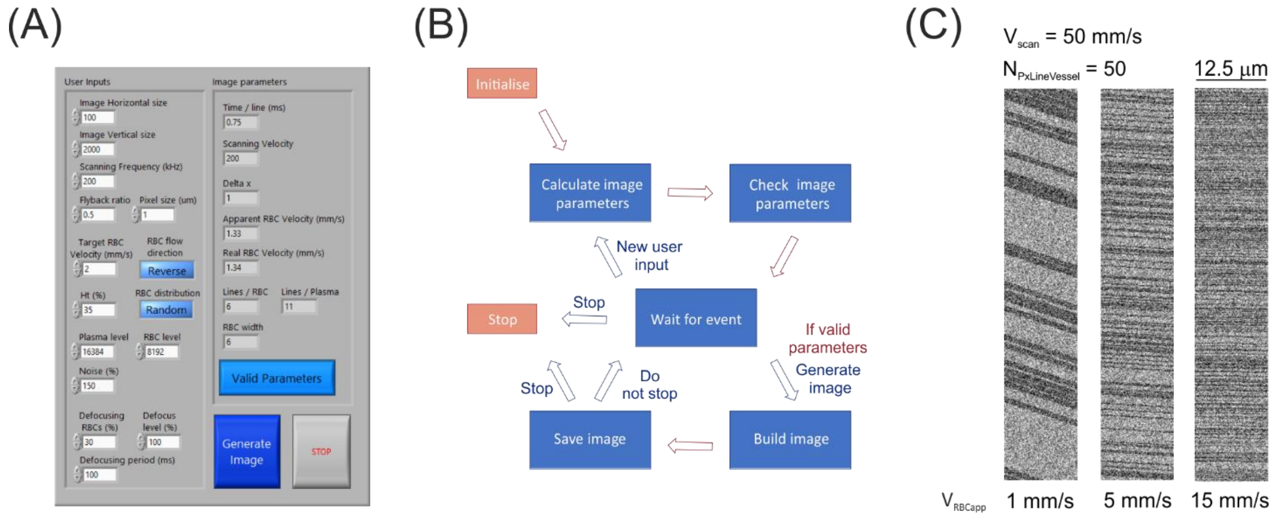

Supplementary Figure 2 : Sofware developped to create artificial linescan images

(A) – Front panel of the software developped to create artificial linescan images showing user-defined input parameters, image parameters which are calculated by the software, and boleans used to show if the parameters are valid and generate images or stop the software.

(B) – State diagram of the software and allowed transitions. Compulsory transitions are represented by red arrows and optional transitions by blue ones. Conditions generating the transitions are indicated next to arrow, using the same color code. Validation of input parameters occurs at the end of the check image parameters state and determines the following state transitions.

(C) – Examples of images created with our software. Each image is 100x1600 pixels. The noise level was set to 150.

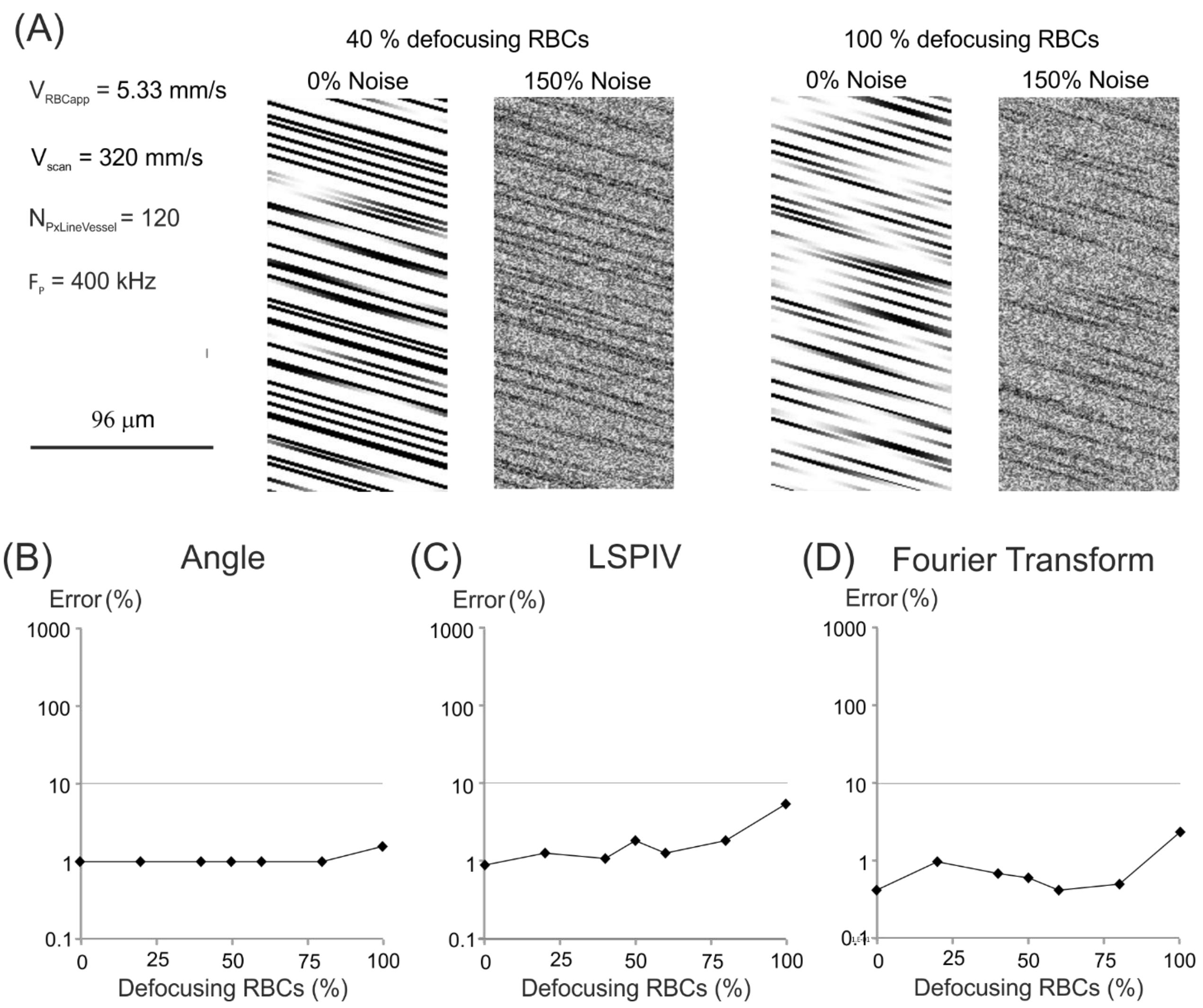

Supplementary Figure 3: Effect of defocusing RBCs on RBC velocity calculation

(A) – Examples of artificial line-scans with defocusing RBCs.

(B-D) – Artificial line-scans of constant velocity (V_{RBCapp}^{image}) with levels of defocusing RBCs ranging from 0 to 100% (see Methods 3.4) were processed with each algorithm and the relative error was calculated (see Methods 3.5).
