# Supplementary figures and images for: Measurement of Blood Velocity With Laser Scanning Microscopy: Modeling and Comparison of Line-Scan Image-Processing Algorithms
Source: Front Physiol. 2022 Apr 7;13:848002. doi: 10.3389/fphys.2022.848002 (PMC9022085; doi:10.3389/fphys.2022.848002)

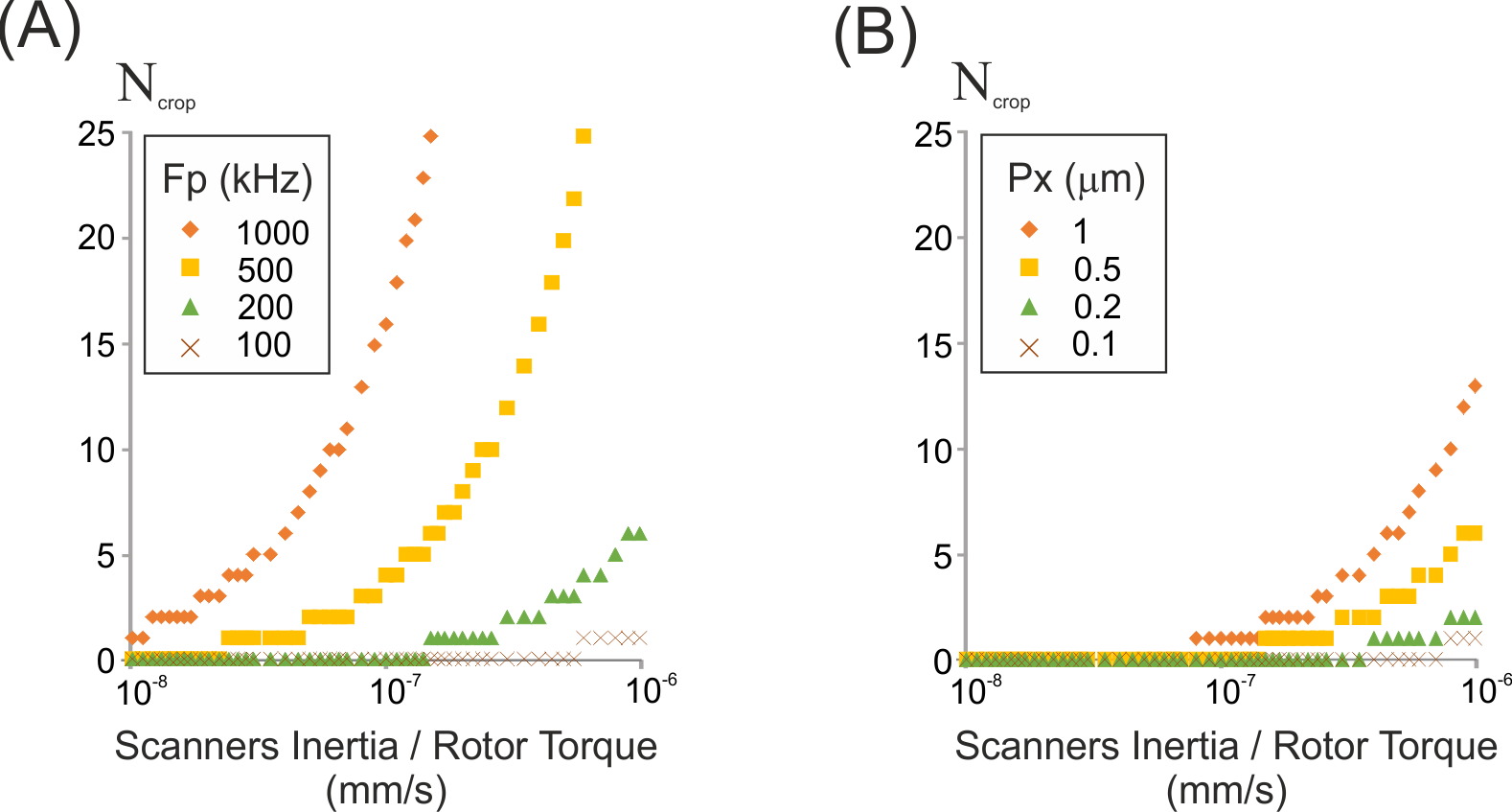

Supplement: Supplementary file 2 [file Image_1.TIF]

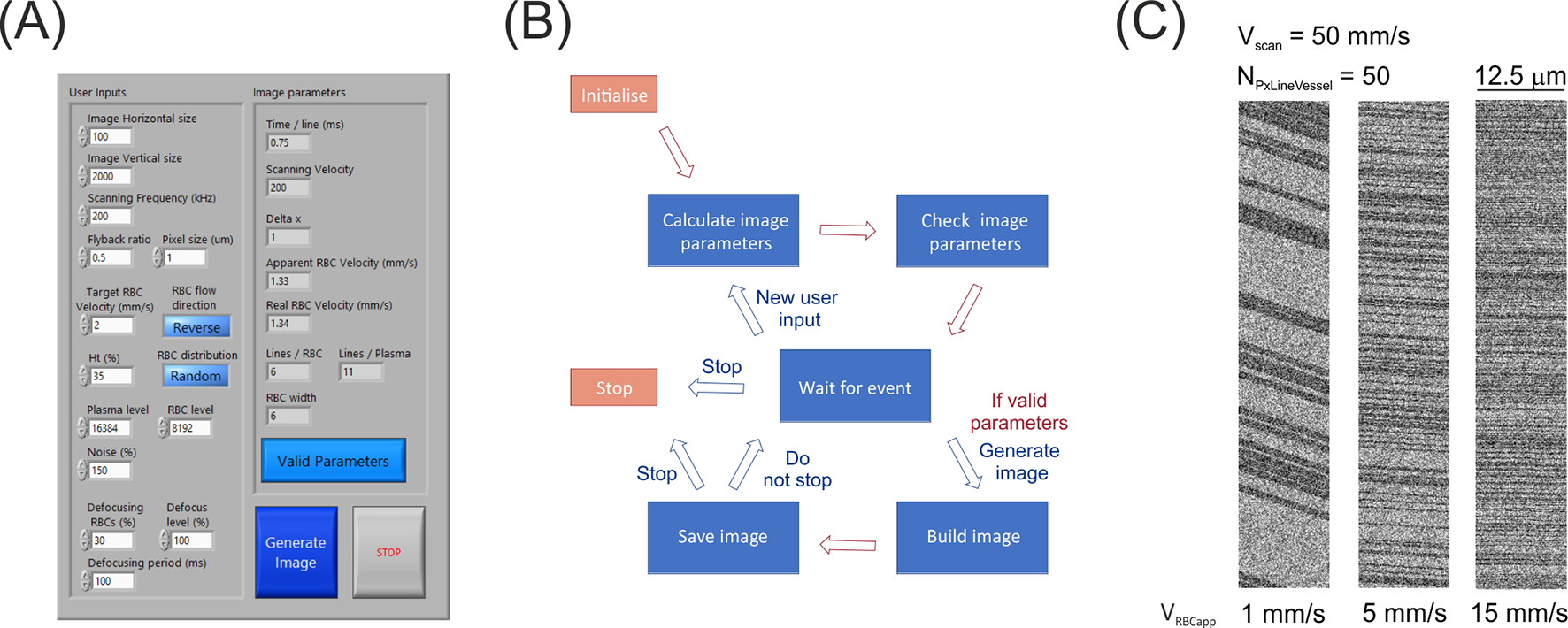

Supplement: Supplementary file 3 [file Image_2.TIF]

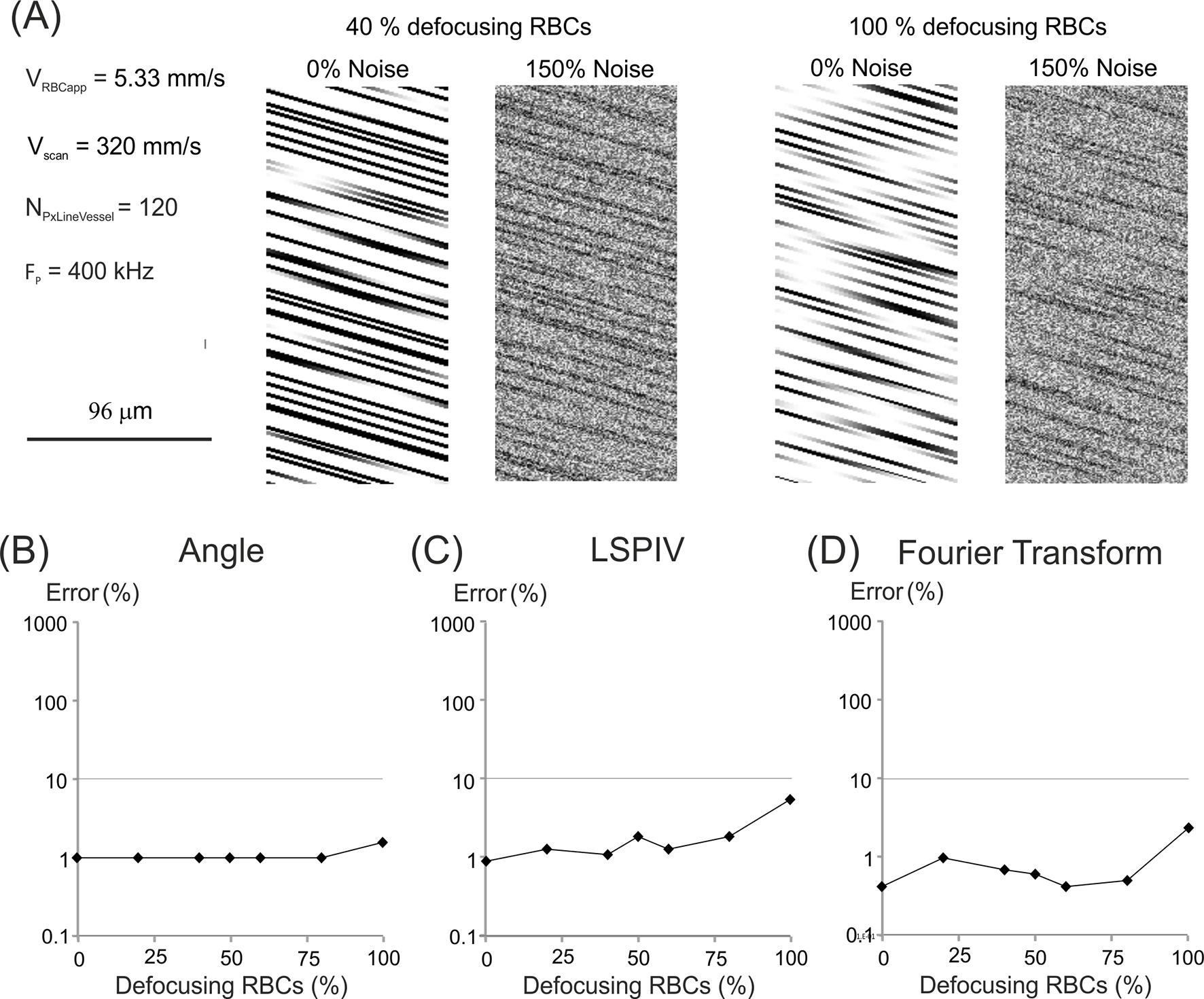

Supplement: Supplementary file 4 [file Image_3.TIF]
